# Apoptosis signal-regulating kinase 1 inhibition attenuates cardiac hypertrophy and cardiorenal fibrosis induced by uremic toxins: Implications for cardiorenal syndrome

**DOI:** 10.1371/journal.pone.0187459

**Published:** 2017-11-06

**Authors:** Feby Savira, Longxing Cao, Ian Wang, Wendi Yang, Kevin Huang, Yue Hua, Beat M. Jucker, Robert N. Willette, Li Huang, Henry Krum, Zhiliang Li, Qiang Fu, Bing Hui Wang

**Affiliations:** 1 Centre of Cardiovascular Research and Education in Therapeutics, Department of Epidemiology and Preventive Medicine, Monash University, Melbourne, Victoria, Australia; 2 Zhujiang Hospital, School of Medicine, Southern Medical University, Guangzhou, China; 3 School of Traditional Chinese Medicine, Southern Medical University, Guangzhou, China; 4 Heart Failure Discovery Performance Unit, GlaxoSmithKline, King of Prussia, Pennsylvania, United States of America; University of Florida College of Medicine, UNITED STATES

## Abstract

Intracellular accumulation of protein-bound uremic toxins in the setting of cardiorenal syndrome leads to adverse effects on cardiorenal cellular functions, where cardiac hypertrophy and cardiorenal fibrosis are the hallmarks. In this study, we sought to determine if Apoptosis Signal-Regulated Kinase 1 (ASK1), an upstream regulator of cellular stress response, mediates cardiac hypertrophy and cardiorenal fibrosis induced by indoxyl sulfate (IS) and *p*-cresol sulfate (PCS) *in vitro*, and whether ASK1 inhibition is beneficial to ameliorate these cellular effects. PCS augmented cardiac myocyte hypertrophy and fibroblast collagen synthesis (as determined by ^3^H-leucine and ^3^H-proline incorporation, respectively), similar to our previous finding with IS. IS and PCS also increased collagen synthesis of proximal tubular cells and renal mesangial cells. Pro-hypertrophic (α-skeletal muscle actin and β-MHC) and pro-fibrotic genes (TGF-β1 and *ctgf*) were induced by both IS and PCS. Western blot analyses revealed the activation of ASK1 and downstream mitogen activated protein kinases (MAPKs) (p38MAPK and ERK1/2) as well as nuclear factor-kappa B (NF-κB) by IS and PCS. ASK1, OAT1/3, ERK1/2 and p38MAPK inhibitors suppressed all these effects. In summary, IS and PCS exhibit pro-hypertrophic and pro-fibrotic properties, at least in part, *via* the activation of ASK1 and its downstream pathways. ASK1 inhibitor is an effective therapeutic agent to alleviate protein-bound uremic toxin-induced cardiac hypertrophy and cardiorenal fibrosis *in vitro*, and may be translated further for cardiorenal syndrome therapy.

## Introduction

Cardiorenal syndrome remains a global challenge, with patients relentlessly faced with high morbidity and mortality rates and significant burden of healthcare-related costs [1]. Due to the complex nature of the disease and the dual involvement of both cardiac and renal systems [2, 3], current therapies have been observed to benefit only one organ to the detriment of the other [4].

Despite recent advances, mechanistic processes involved in pathophysiological changes leading to CRS progression are still poorly understood [5]. At the cellular level, cardiac hypertrophy as well as cardiac and renal fibrosis are the hallmarks of pathological changes within the heart and the kidneys. Excessive activation of these cellular processes is critical in mediating both cardiac and renal impairment, contributing to CRS pathophysiology.

One distinct feature of chronic kidney disease (CKD) is systemic retention and accumulation of uremic toxins [6]. Indoxyl sulfate (IS) and p-cresol sulfate (PCS) are extensively studied due to their tendency to bind to albumin, thereby forming large protein complexes that are undialyzable through the pores of the dialysis membrane [6, 7]. In dialysis patients, serum levels of IS and PCS are elevated by 54 and 17 times, respectively, whereas their amounts are undetectable in healthy individuals [5]. Both toxins are associated with increased mortality in patients with cardiovascular disease (CVD) and renal impairment [8–10]. Targeting these toxic solutes and related pathways may be of a high therapeutic value to attenuate CRS progression [11].

Apoptosis Signal-Regulating Kinase 1 (ASK1) pathway is part of the mitogen-activated protein kinase kinase kinase (MAP3K or MEKK) family, involved in an array of cellular stress responses leading to apoptosis [12]. ASK1 has been implicated in the progression of various diseases, including heart and kidney dysfunction [13, 14]. The pathological role of ASK1 in these organs mainly involves reactive oxygen species (ROS) production [14].

While many reports have shown increased ROS production after exposure to protein-bound uremic toxins [7], the activation of ASK1, a ROS-sensitive kinase, is essentially unproven in such setting. We have previously reported that IS induces hypertrophy and collagen synthesis of cardiac cells *via* the activation of ERK1/2, p38MAPK and NF-κB pathways[11], which lie downstream within the ASK1 signaling cascade [15, 16]. In this study, we sought to determine the direct effects of PCS on cardiac myocyte hypertrophy and cardiac fibroblast collagen synthesis, as well as collagen synthesis of renal cells induced by IS and PCS, along with upregulation of pro-hypertrophic and pro-fibrotic genes. We also attempted to delineate the role of ASK1 and its downstream pathways in mediating these cellular effects and whether the inhibition of ASK1 is beneficial to ameliorate cardiac and renal cellular remodeling induced by IS and PCS in an *in vitro* setting.

## Methods

### Materials

IS and PCS were acquired from Sigma-Aldrich (St. Louis, MO, USA). Stock solution of both IS and PCS were prepared with sterilized and endotoxin-free phosphate-buffered saline (PBS) and stored in -20°C until use. The selective ASK1 inhibitor GSK2261818A (G226) was a gift received from GlaxoSmithKline (GSK)(Heart Failure Discovery Performance Unit, King of Prussia, PA, USA). The enzyme inhibition activity for ASK1 is pKi 7.70 with more than 20 fold selectivity over 15 other related and unrelated kinases, which have been tested by GSK. These results showed that the agent is a good tool compound for proof of concept studies such as this *in vitro* study. ERK1/2 upstream inhibitor (MEK1/2 inhibitor, U0126) (Sigma-Aldrich) and p38MAPK inhibitor (RWJ-67657) were kind gifts from Scott Wadsworth (Johnson & Johnson Pharmaceutical Research & Development, L.L.C.). As demonstrated previously, RWJ-67657 and U0126 is highly selective for p38 (α and β) [17] and MEK1/2 (ERK1/2 upstream) [18], respectively. Both RWJ-67657 and U0126 have been widely used to inhibit p38MAPK and ERK1/2 in various disease settings. Probenecid is a potent OAT1/3 inhibitor mainly used for the treatment of gout in the clinic [6] and has been extensively used to study renal-related functions as well as its functional relevance with uremic toxins such as IS and PCS [19–21]. The stock solution of G226, U0126, RWJ-67657 and Probenecid was prepared in dimethyl sulfoxide and kept in -20°C until needed. Other reagents were purchased from Sigma.

### Culture of cardiac and renal cells

Neonatal rat cardiac myocyte (NCM) and fibroblast (NCF) were isolated by enzymatic digestion from neonatal Sprague-Dawley rat pups aged 1 to 2-days old as detailed previously [11]. The Alfred Medical Research and Education Precinct Animal Ethics Committee approved the animal use for this study (approval no. E/0980/2010/M). The protocol used complies with the guidance from the National Health and Medical Research Council of Australia in the care and use of laboratory animals. Briefly, rat pups were sacrificed by decapitation and NCMs and NCFs were extracted from the isolated hearts by enzyme digestion as previously described [22, 23]. NCMs were seeded in MEM containing 10% NBCS and 0.1 mM BrDu at a density of 300,000 cells per well in 12-well plates and maintained in serum-free DMEM supplemented with insulin, apo-transferrin and 50 mM KCl. BrDu was only used for the first three days. KCl was added to hinder contact-induced spontaneous contraction of myocytes. NCFs were initially seeded into T75 flasks and maintained in high-glucose DMEM containing 1% antibiotic/antimycotic and 10% Fetal Bovine Serum (FBS; JRH Biosciences, Lenexa, KA, USA).

Rat renal mesangial cells (RMC) and human kidney-2 (HK2) proximal tubular cells were purchased from the American Type Culture Collection (ATCC) (RMC: ATCC CRL-2573, HK2: ATCC CRL-2190) and cultured according to the protocol provided by ATCC.

### Measurement of neonatal rat cardiac myocyte hypertrophy

^3^H-leucine incorporation was used to determine NCM hypertrophy as described previously [11]. NCMs were pre-treated with or without selective ASK1 inhibitor (G226, 0.03 to 1.0 μM), p38MAPK inhibitor (RWJ-67657, 0.1 to 3.0 μM), ERK1/2 inhibitor (U0126, 0.03 to 1.0 μM) and OAT1/3 antagonist (Probenecid, 0.1 to 100.0 μM) for 2 hours. IS and PCS were added at a concentration of 10 and 100 μM, respectively. These doses were used for each inhibitor after dosage optimization studies validated their effectiveness (data not shown). 1 μCi of ^3^H-leucine was added to each well. NCMs were incubated for 48 hours before harvested by 10% trichloroacetic acid (TCA) precipitation on ice for 30 minutes and solubilization with 1M NaOH overnight at 4°C. 1M HCl was used to neutralize the samples, and the levels of ^3^H-leucine incorporations were determined on a beta counter after re-suspension in scintillation fluid.

### Measurement of cardiac fibroblast, renal mesangial cell and proximal tubular cell collagen synthesis

Collagen synthesis of NCF, RMC and HK2 cell was measured by ^3^H-proline incorporation. NCFs were maintained and used at passage 2 and seeded at a density of 50,000 cells per well in 12-well plates and serum starved for 48 hours in DMEM supplemented with 1% vitamin C and 0.5% Bovine Serum Albumin (BSA). RMCs and HK2 cells were cultured and maintained until ~80% confluence before seeded for treatment. RMCs were seeded at a density of 4,000 cells per well in 12-well plates and maintained in high glucose DMEM containing 15% FBS and 1% antibiotic/antimycotic. HK2 cells were seeded into 12-well plates at a density of 5,000 cells per well and maintained in Keratinocyte Serum Free Medium (KSFM) containing 5 ng/ml Epidermal Growth Factor (EGF), 0.05 mg/ml Bovine Pituitary Extract (BPE) and 10% FBS in the presence of 1% antibiotic/antimycotic. For all cell types, this was followed by serum starvation with 0.5% BSA for 48 hours prior to treatment.

NCFs and RMCs were pre-treated for 2 hours with or without selective ASK1 inhibitor (G226, 0.03 to 1.0 μM), p38MAPK inhibitor (RWJ-67657, 0.1 to 3.0 μM), ERK1/2 inhibitor (U0126, 0.03 to 1.0 μM) and OAT1/3 antagonist (Probenecid, 0.1 to 100.0 μM) before stimulation with either IS or PCS (10 and 100 μM, respectively). HK2 cells were pre-treated (2 hours) with or without G226 at a concentration range of 0.1 to 3.0 μM, followed by the addition of IS or PCS. ^3^H-proline (1 μCi) was added into each well and all cells were further incubated for 48 hours before harvest by 10% TCA precipitation (30 minutes), 1M NaOH solubilization (overnight at 4°C), neutralization with 1M HCl and resuspension in scintillation fluid to measure the levels of ^3^H-proline incorporation with beta counter.

### Western blot analysis

NCMs were seeded in 6-well plates at a density of 1x10^6^ cells per well, while RMCs were seeded in T75 flasks with a density of 1x10^6^ cells per flask. All cells were serum starved on the following day for 48 hours similar to hypertrophy and collagen synthesis assays described above. Subsequently, cells were pre-treated for 2 hours with or without selective ASK1 inhibitor (G226, 1.0 μM), p38MAPK inhibitor (RWJ-67657, 3.0 μM), ERK1/2 inhibitor (U0126, 1.0 μM) and OAT1/3 antagonist (Probenecid, 100.0 μM) followed by stimulation by either IS (10 μM) or PCS (100 μM) for 15 minutes. Cells were then lysed with ~50–80 μl of modified RIPA lysis buffer containing protease and phosphatase inhibitors. Protein concentration of the sample was measured by Bradford assay. Equal amount of protein samples (10 or 20 μg) were separated by SDS polycramide gel electrophoresis (SDS-PAGE) and transferred onto a nitrocellulose membrane (Amersham Hybond ECL, GE Healthcare, Freiburg, Germany) by electrophoresis. Western blot analysis was conducted according to the manufacturer’s protocol using specific primary antibodies (phospho-ASK1, phospho-p38, p38, phospho-ERK1/2, ERK1/2, phospho-NF-κB p65, NF-κB p65 and Pan Actin; see S1 Table for full specification of antibodies used). Protein bands were visualized with Super Signal West Pico Chemiluminescence Substrates (Thermo Fisher Scientific, Rockford, IL, USA). Analysis of band intensity was performed on ImageJ software (National Center for Biotechnology Information). Please refer to S1 to S6 Figs for the full membrane image of each primary antibody.

### Quantitative measurement of pro-hypertrophic and pro-fibrotic gene expression in cardiac myocyte and fibroblast

NCMs, NCFs and HK2 cells were seeded at the following densities, respectively: 500,000 cells, 200,000 cells and 50,000 cells per well in 6-well plates and serum starved as specified above. After pre-treatment with or without selective ASK1 inhibitor (1.0 μM), Pro (1.0 μM), RWJ-67657 (3.0 μM) and U0126 (1.0 μM) for 2 hours and stimulation with either IS (10 μM) or PCS (100 μM), the cells were incubated in 5% CO_2_ at 37°C for 18 hours. Cells were then harvested and total RNA extraction was performed using MagMAX-96 Total RNA Isolation for Microarray Kit according to the manufacturer’s protocol (Thermo Fisher Scientific, Rockford, IL, USA). Reverse transcription of mRNA into cDNA was performed with MultiScribe (Applied Biosystems, Foster City, CA, USA). Amplification of triplicate cDNA aliquots (1 μl) was performed using sequence-specific primers (Geneworks, Adelaide, SA, Australia) with SYBR Green detection (Applied Biosystems). The expression of pro-hypertrophic (α-skeletal muscle actin (α-SkM-Ac) and β-MHC) and pro-fibrotic-related markers (TGF-β1 and *ctgf*) were quantified by real-time polymerase chain reaction (PCR) on the QuanStudio 12K Flex Real Time PCR System (Applied Biosystems). Primer Express 2.0 (Applied Biosystems) software was utilized to design the primers for rat cell lines based on sequences published by NCBI (http://www.ncbi.nlm.nih.gov). The sequence of human primers of *ctgf* and TGF-β1 were obtained based on previously published studies. Full list of primer sequence can be found in S2 Table. GAPDH was used as endogenous controls for NCMs and NCFs and 18S rRNA for HK2 cells.

### Measurement of cardiac and renal cell viability

Cells were seeded in 96-well plates at the following densities: 15,000 for NCFs and 3,000 for HK2 cells. After 48 hours of serum starvation, NCFs were stimulated with PCS at a concentration range of 0.0001 to 100 μM for a further of 48 hours. HK2 cells and NCFs were pre-treated with or without the selective ASK1 inhibitor (G226, 0.1 to 3.0 μM) for 2 hours before stimulation with either IS or PCS (10 or 100 μM, respectively) and incubated further for 48 hours. The 3-(4,5-dimethyl-2 thiazoyl)-2,5-diphenyl-2H-tetrazolium bromide (MTT) assay was then performed to determine cell viability as described previously [11].

### Statistical analysis

Cell culture experiments (hypertrophy and collagen synthesis assays) were performed in triplicate and repeated for at least three times. Cell viability assays were performed in quadruplicates for each condition and repeated two to three times. The results are presented as the percentage of unstimulated controls (mean ± SEM). For Western blot analyses, preliminary experiments were performed to determine the activation of pathways and then repeated with additional experiments in triplicates for each condition. Ratio of phosphorylated over total protein levels were analyzed (except for ASK1 where normalization was done with Pan Actin). For real-time PCR, gene expression levels in NCF and NCM were normalized with GAPDH (housekeeping gene) and 18S for HK2 cells, all in triplicates. One-way ANOVA with Bonferroni’s multiple comparison post hoc tests was used for statistical analyses for comparison between multiple groups and unpaired t-test was used for comparison between two groups. A statistically significant result was determined with a two-tailed *p*-value of less than 0.05. The software used to perform all of the statistical analyses was GraphPad Prism Version 7 (GraphPad Software Inc., USA).

## Results

### Direct effects of indoxyl sulfate and *p*-cresol sulfate on cardiac myocytes and fibroblasts

**Fig 1:**
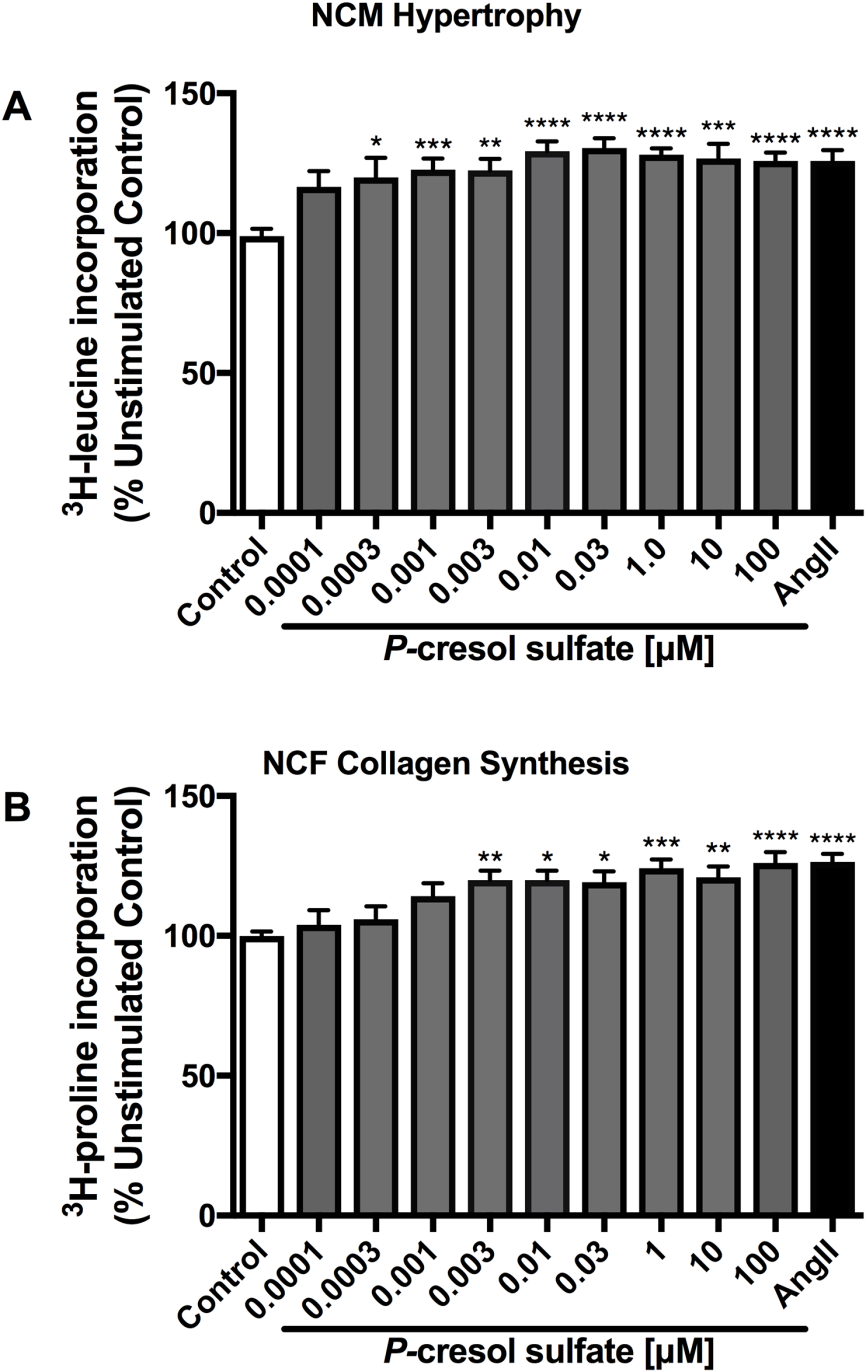
Effects of *p*-cresol sulfate on cardiac myocyte hypertrophy and fibroblast collagen synthesis. PCS significantly induced NCM hypertrophy starting from the lowest dose (0.001 μM, *p*<0.01 vs control) (A) and NCF collagen synthesis (B) starting at 0.003 (*p*<0.01 vs control). Ang-II has been included as a positive control. Data are presented as mean ± SEM of triplicates from three experiments. *p<0.05, **p<0.01, ***p<0.001, ****p<0.0001 vs control, One-way Anova.

Stimulation with PCS at concentrations of 0.001 to 100 μM significantly augmented NCM hypertrophy (Fig 1A) as determined by ^3^H-leucine incorporation as well as NCF collagen synthesis at concentrations of 0.003 to 100 μM as determined by ^3^H-proline incorporation (Fig 1B). The lowest effective dose of PCS on NCM (0.001 μM) induced a 122.6% increase in hypertrophy, while in NCF (0.003 μM of PCS), a 119.9% increase in cellular collagen synthesis was observed. Ang-II was used as a positive control.

**Fig 2:**
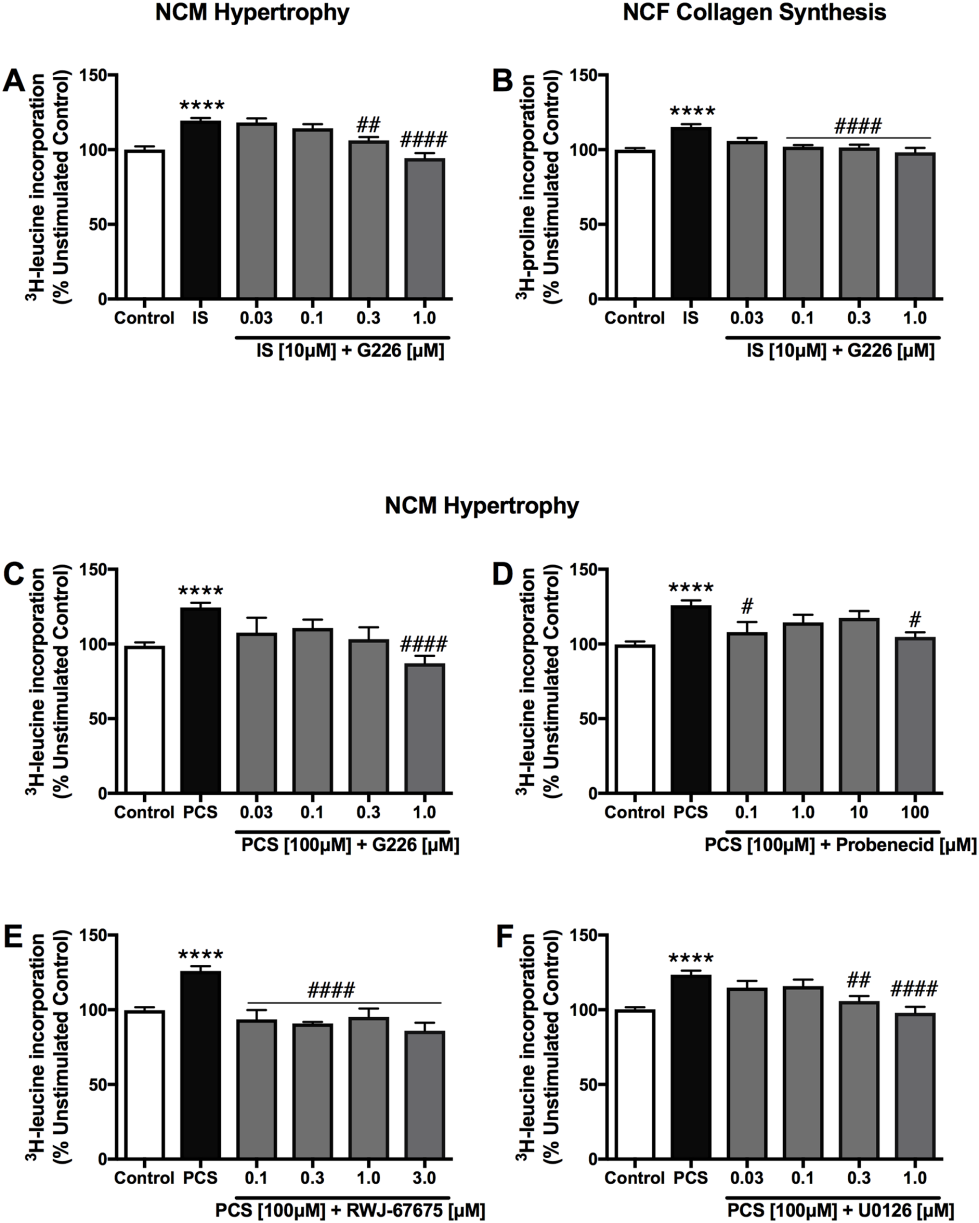
Effects of selective ASK1, OAT1/3, p38MAPK and ERK1/2 inhibitors on indoxyl sulfate- and *p*-cresol sulfate-stimulated cardiac cells. Both NCM hypertrophy (A) and NCF collagen synthesis (B) induced by IS (10 μM) were dose-dependently abrogated by G226, indicating the involvement of ASK1 pathway in cardiac cellular remodeling induced by IS. Hypertrophy of NCM by stimulated by PCS (100 μM) were also significantly attenuated by G226 (C), Probenecid (D), RWJ-67657 (E) and U0126 (F), suggesting the role of OAT1/3-ASK1-MAPK cascade in PCS-induced cardiac remodeling. Data are presented as mean ± SEM from three different experiments, each with triplicates. ****p<0.0001 IS [10 μM] or PCS [100 μM] vs control, ^#^*p*<0.05, ^##^*p*<0.01, ^####^*p*<0.0001 vs IS [10 μM] or PCS [100 μM], One-way Anova.

IS (10 μM) directly stimulated NCM hypertrophy and NCF collagen synthesis (by 119.4% and 115.2% vs. control, respectively, *p*<0.0001). The selective ASK1 inhibitor, G226, dose-dependently reduced these effects by IS (10 μM) at a concentration range of 0.03 to 1.0 μM (Fig 2A and 2B).

In addition, dose-dependent inhibitions were also seen in PCS-stimulated NCM hypertrophy (100 μM) after co-treatment with G226 (Fig 2C), OAT1/3 antagonist (Probenecid) (Fig 2D) as well as p38MAPK and ERK1/2 inhibitors (RWJ-67657 and U0126, respectively) (Fig 2E and 2F) at indicated concentrations.

### Direct effects of *p*-cresol sulfate on cardiac cells occur *via* ASK1 and MAPK pathways

**Fig 3:**
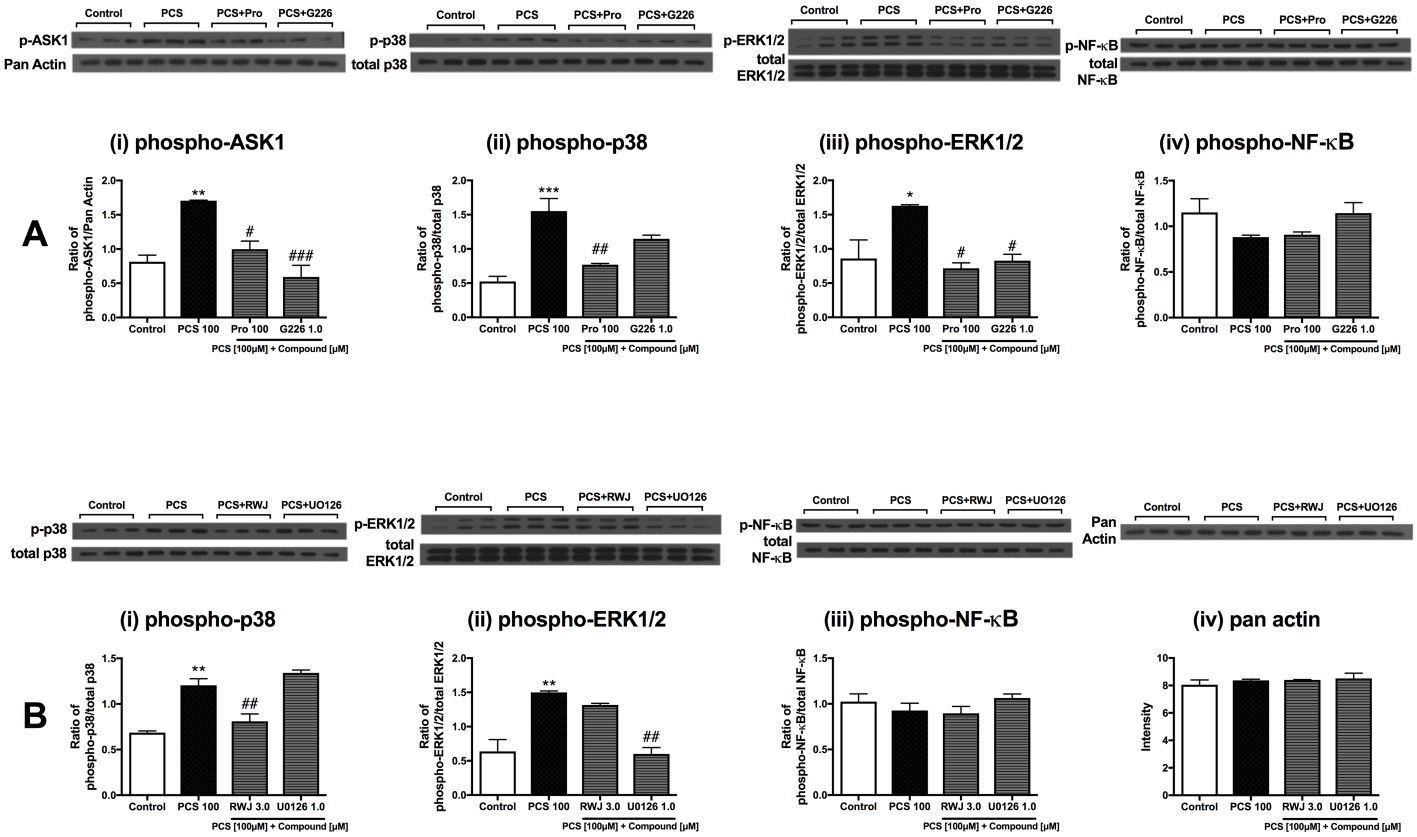
Signaling pathway activation in *p*-cresol sulfate-stimulated neonatal cardiac myocytes. Representative images and quantification of Western blot analyses of NCMs stimulated with PCS in the absence and presence of (A) 100 μM Probenecid and 1.0 μM G226 for (i) phospho-ASK1, (ii) phospho-p38, (iii) phospho-ERK1/2, (iv) phospho-NF-κB; and (B) 3.0 μM RWJ-67657 and 1.0 μM U0126 for (i) phospho-p38, (ii) phospho-ERK1/2, (iii) phospho-NF-κB and (iv) Pan Actin. Data are presented as mean ± SEM (n = 3). *p<0.05, **p<0.01, ***p<0.001 vs control, ^#^*p*<0.05, ^##^*p*<0.01, ^###^*p*<0.001 vs PCS [100 μM], One-way Anova.

PCS stimulated activation of ASK1, p38MAPK and ERK1/2 pathways in NCMs as determined by significantly elevated phosphorylation levels of these proteins (Fig 3). However, phospho-NF-κB protein level did not increase. Co-treatment with Probenecid (100 uM) and G226 (1.0 uM) reduced levels of phospho-ASK1 and downstream phospho-ERK1/2 and phospho-p38MAPK (Fig 3A). RWJ-67657 (3.0 uM) and U0126 (1.0 uM) significantly suppressed p38MAPK and ERK1/2 activation, respectively (Fig 3B). These results also support the selectivity of these compounds, where G226 was able to inhibit ASK1 and downstream MAPKs (p38MAPK and ERK1/2), while RWJ-67657 and U0126 exclusively inhibit p38MAPK and ERK1/2, respectively, as intended.

### Indoxyl sulfate and *p*-cresol sulfate augmented collagen synthesis of renal mesangial cells and proximal tubular cells

**Fig 4:**
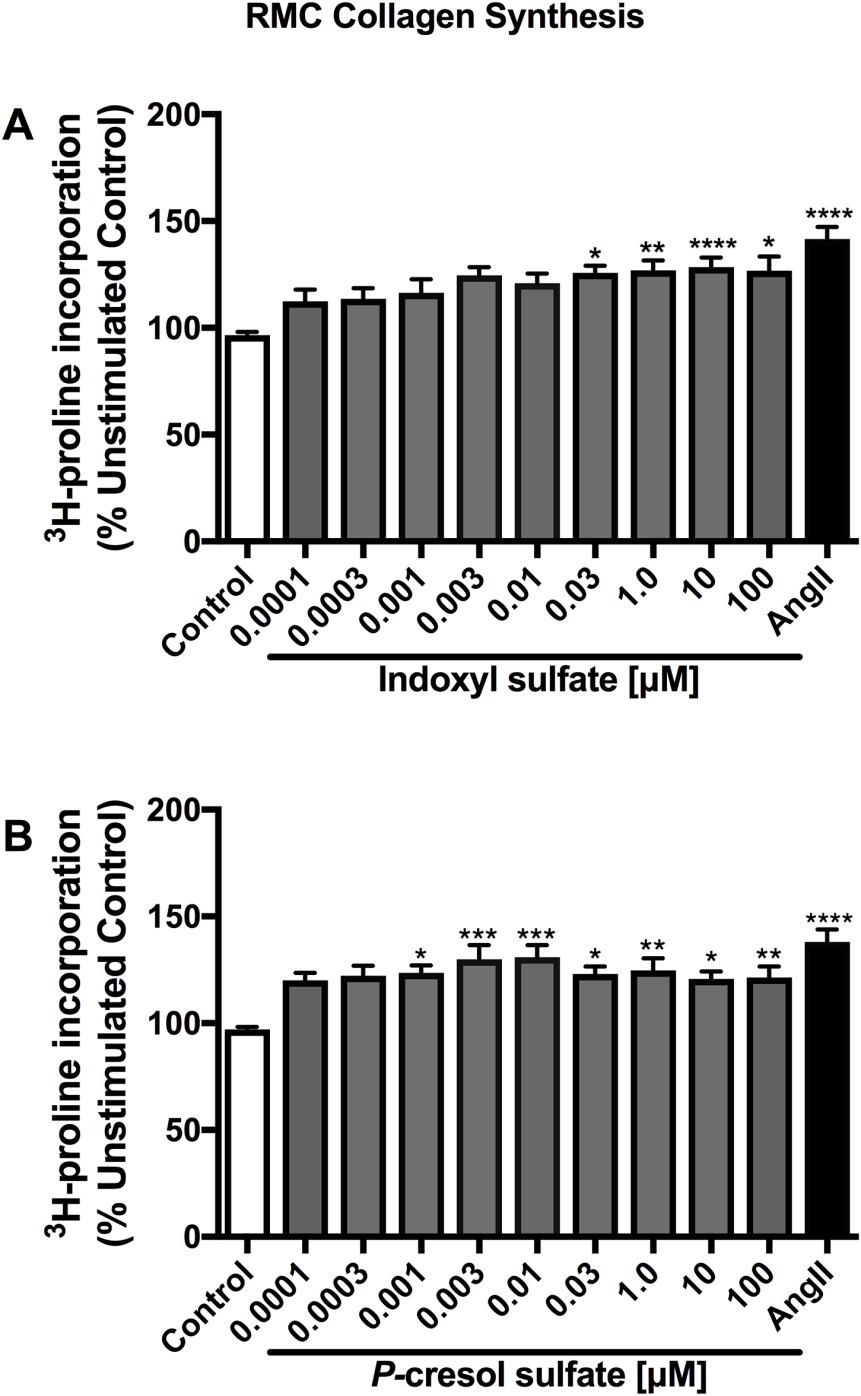
Effects of indoxyl sulfate and *p*-cresol sulfate on renal mesangial cell collagen synthesis. IS (A) significantly augmented RMC collagen synthesis at a concentration range of 0.03 to 100 μM, while the effect by PCS (B) was seen in as low as 0.001 μM (*p*<0.05 vs control). Ang-II has been included as a positive control. Data are presented as mean ± SEM from three independent experiments in triplicates. *p<0.05, **p<0.01, ***p<0.001, ****p<0.0001 vs control, One-way Anova.

**Fig 5:**
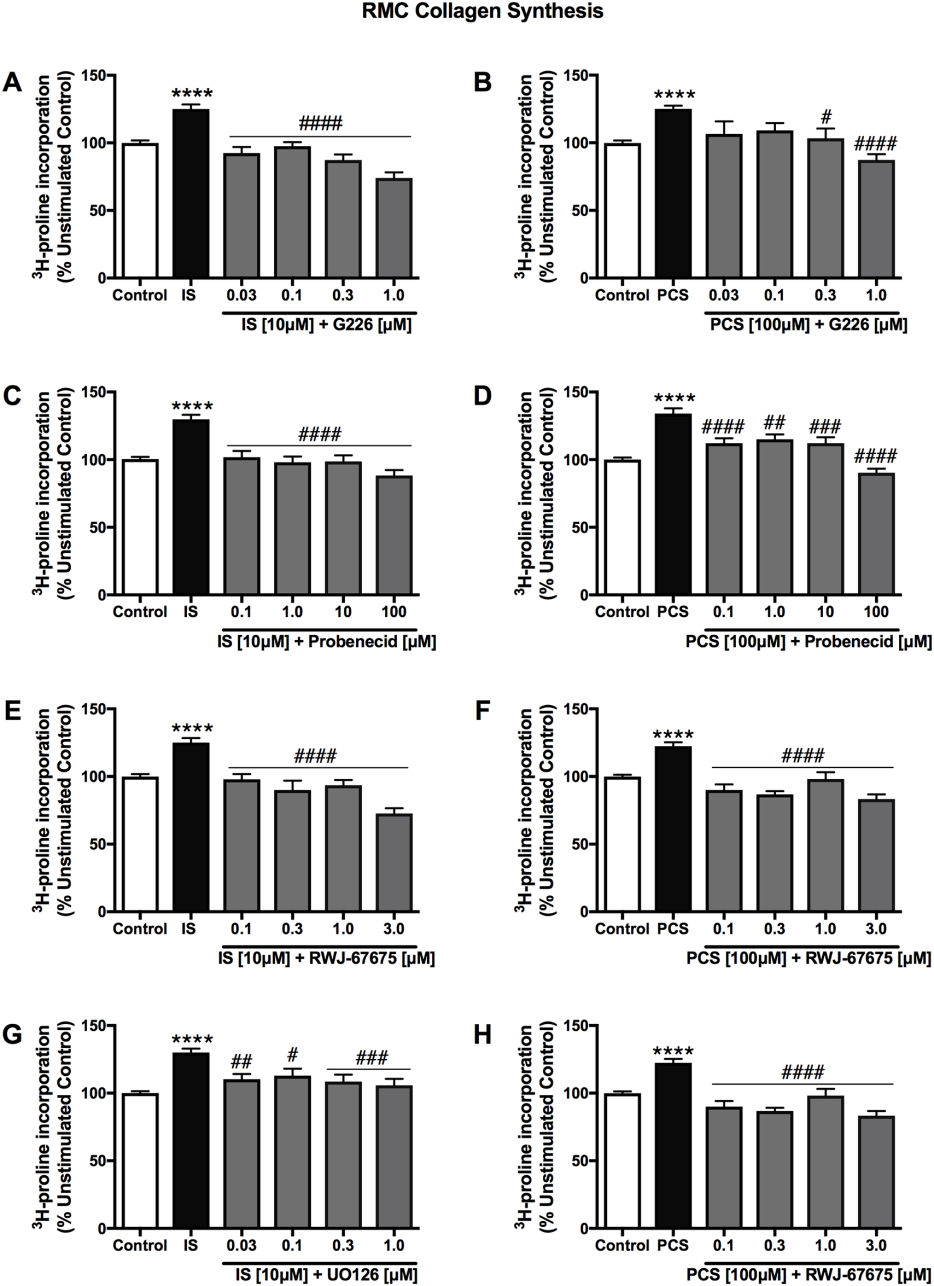
Effects of selective ASK1, OAT1/3, p38MAPK and ERK1/2 inhibitors on indoxyl sulfate- and *p*-cresol sulfate-stimulated renal mesangial cells. Levels of collagen synthesis in both IS- and PCS-stimulated RMCs were dose-dependently reduced by G226 (A and B), Probenecid (C and D), RWJ-67657 (E and F) and U0126 (G and H) back to baseline levels or lower. Data are presented as mean ± SEM from the triplicates of three independent experiments. ****p<0.0001 IS [10 μM] or PCS [100 μM] vs control, ^#^*p*<0.05, ^##^*p*<0.01, ^###^*p*<0.001, ^####^*p*<0.0001 vs IS [10 μM] or PCS [100 μM], One-way Anova.

IS (0.03 to 100 μM) and PCS (0.001 to 100 μM) significantly increased collagen synthesis of RMCs as determined by ^3^H-proline incorporation (Fig 4A and 4B). At the lowest effective dose, IS (0.03 μM) and PCS (0.03 μM) induced a 125.8% and a 123.5% (*p*<0.05) elevation of RMC collagen synthesis compared to control, respectively. Pre-treatment with selective ASK1, OAT1/3, p38MAPK and ERK1/2 inhibitors for 2 hours dose-dependently attenuated the collagen synthesis in RMCs stimulated by IS (10 μM) and PCS (100 μM) (Fig 5C–5H).

**Fig 6:**
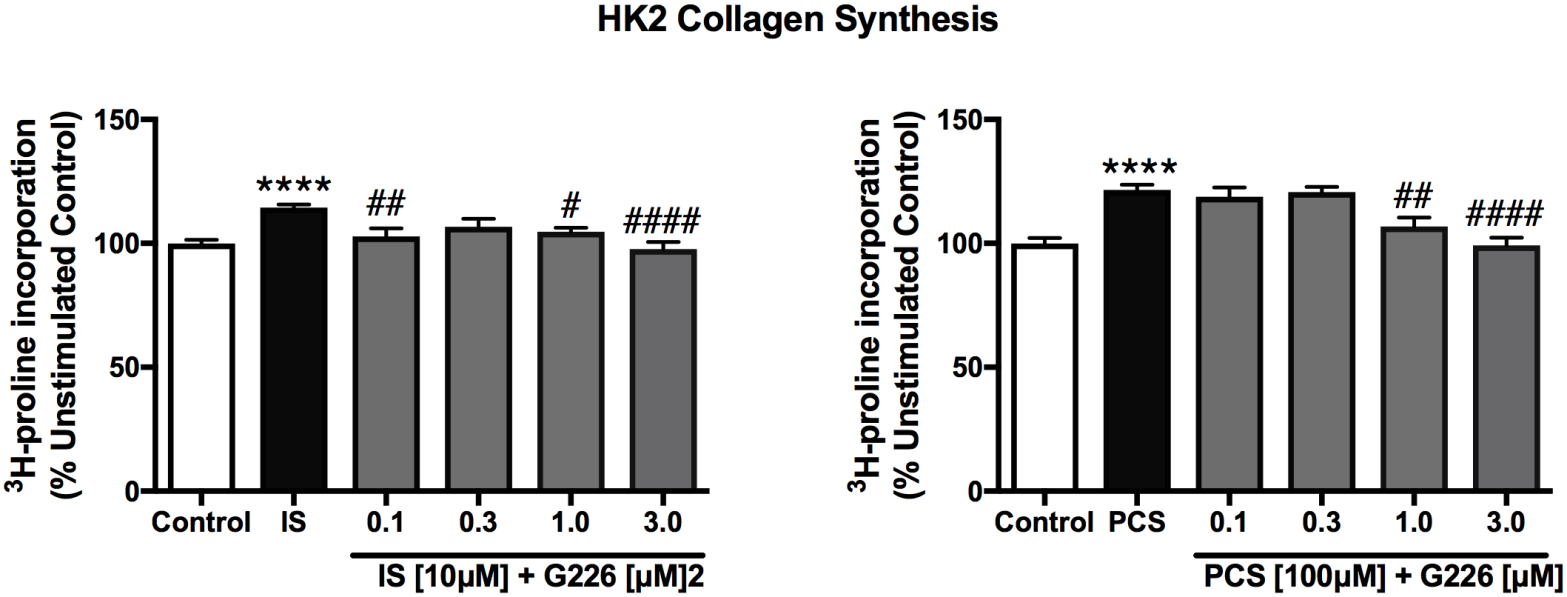
Effects of indoxyl sulfate and *p*-cresol sulfate stimulation with and without selective ASK1 inhibitor (G226) treatment on HK2 cell collagen synthesis. IS (10 μM) and PCS (100 μM) augmented collagen synthesis of HK2 cells. Administration of G226 attenuated IS- and PCS-stimulated collagen synthesis in HK2 cells in a dose-dependent manner. Data are presented as mean ± SEM from three experiments with triplicates. ****p<0.0001 IS [10 μM] or PCS [100 μM] vs control, ^#^*p*<0.05, ^##^*p*<0.01, ^####^*p*<0.0001 vs IS [10 μM] or PCS [100 μM], One-way Anova.

IS (10 μM) and PCS (100 μM) triggered a 114.4% and a 121.6% elevation in HK2 cell collagen synthesis, respectively, compared to unstimulated control (*p*<0.0001) (Fig 6). The effect of IS and PCS was slightly greater in RMCs at these concentrations, where both induced collagen synthesis 25% higher than unstimulated control (*p*<0.0001) (Fig 5A and 5B). These effects were abrogated by G226 (0.1 to 3.0 μM) in a dose-dependent manner.

### Direct effects of indoxyl sulfate and *p*-cresol sulfate on renal cells occur *via* ASK1, MAPK and NF-kB pathways

**Fig 7:**
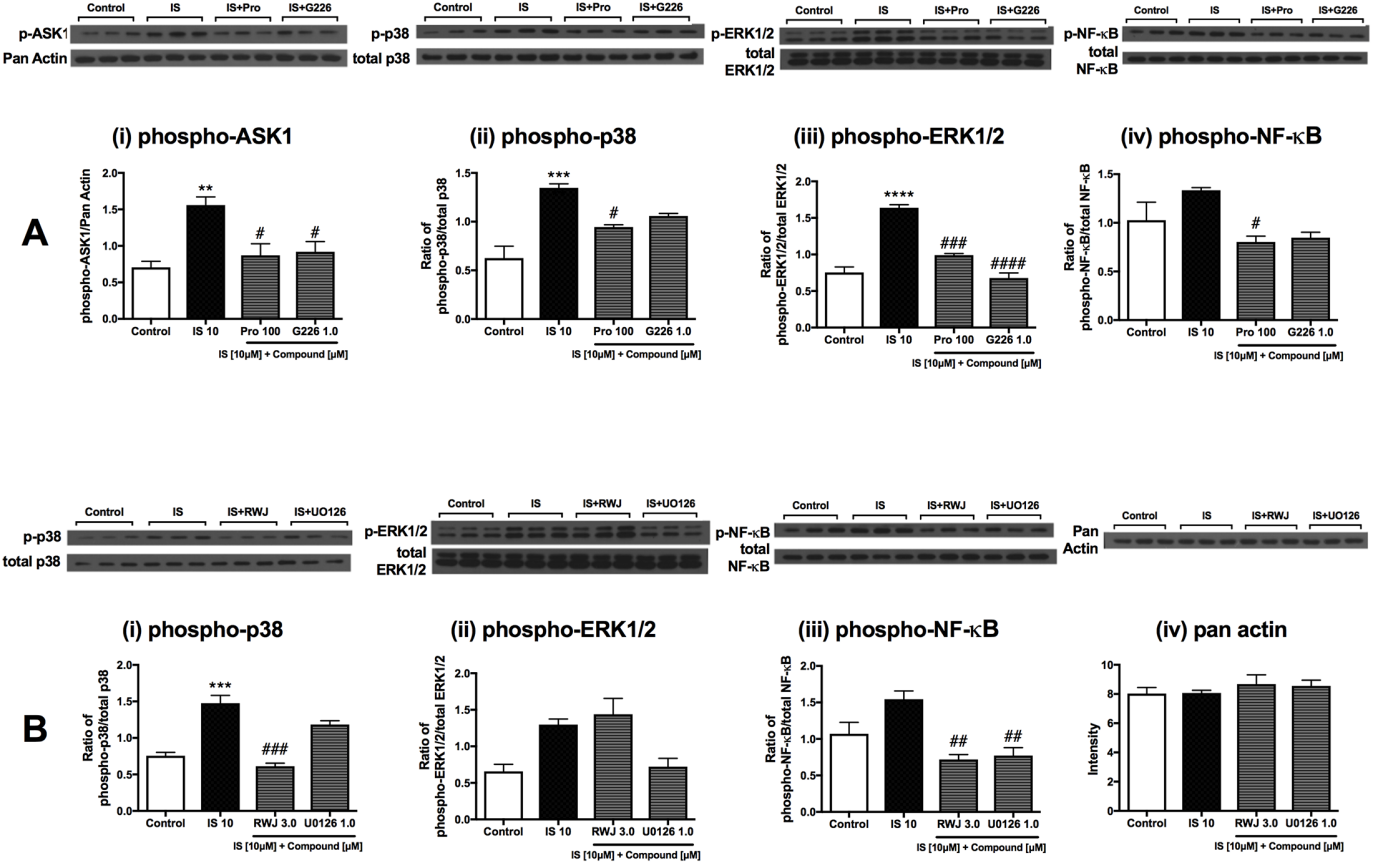
Signaling pathway activation in indoxyl sulfate-stimulated renal mesangial cells. Representative images and quantification of Western blot analyses of RMCs stimulated with IS and co-treated with (A) 100 μM Probenecid and 1.0 μM G226 for (i) phospho-ASK1, (ii) phospho-p38 (^##^*p* = 0.0014 G226 [1.0 μM] vs IS [10 μM], unpaired t-test), (iii) phospho-ERK1/2, (iv) phospho-NF-κB (^##^*p* = 0.0014 G226 [1.0 μM] vs IS [10 μM], unpaired t-test); and (B) 3.0 μM RWJ-67657 and 1.0 μM U0126 for (i) phospho-p38, (ii) phospho-ERK1/2 (**p = 0.0063 IS [10 μM] vs control, ^##^*p* = 0.0136 U0126 [1.0 μM] vs IS [10 μM], unpaired t-test), (iii) phospho-NF-κB and (iv) Pan Actin. Data are presented as mean ± SEM (n = 3). **p<0.01, ****p<0.0001 vs control, ^#^*p*<0.05, ^##^*p*<0.01, ^####^*p*<0.0001 vs IS [10 μM], One-way Anova.

Normalization with endogenous control (pan-Actin) indicated that ASK1 was markedly activated by IS after stimulation for 15 minutes (Fig 7A, i) in RMCs. In addition, the levels of downstream kinases, phospho-p38MAPK and phospho-ERK1/2, were also significantly elevated (Fig 7A, ii & iii). NF-κB activation was also apparent but not statistically significant (Fig 7A, iv). Probenecid (100 μM) and G226 (1.0 μM) abrogated phosphorylation levels of these pathways (Fig 7A, i-iv).

RWJ-67657 (3.0 μM) and U0126 (1.0 μM) attenuated phospho-p38MAPK and phospho-ERK1/2 levels, respectively, and both inhibitors also markedly abrogated downstream NF-κB activation (Fig 7B, i-iii). Pan-Actin levels were unchanged (Fig 7B, iv).

**Fig 8:**
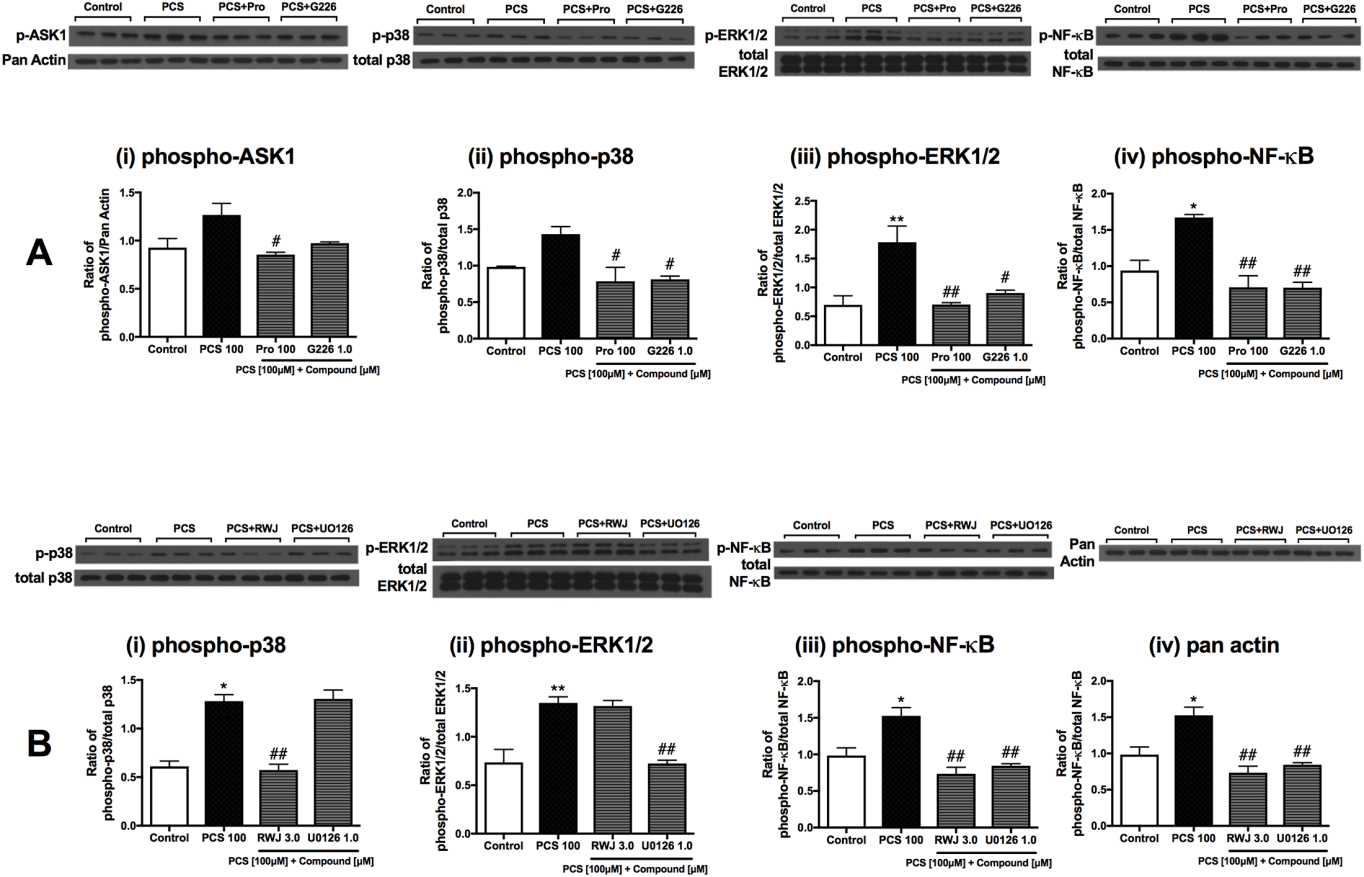
Signaling pathway activation in *p*-cresol sulfate-stimulated renal mesangial cells. (A) Representative images of the Western blot analyses of RMCs stimulated with PCS and co-treated with (A) 100 μM Probenecid and 1.0 μM G226 for (i) phospho-ASK1, (ii) phospho-p38 (*p = 0.012 IS [10 μM] vs control, unpaired t-test), (iii) phospho-ERK1/2, (iv) phospho-NF-κB; and (B) 3.0 μM RWJ-67657 and 1.0 μM U0126 for (i) phospho-p38, (ii) phospho-ERK1/2, (iii) phospho-NF-κB and (iv) Pan Actin. Data are presented as mean ± SEM (n = 3). *p<0.05, **p<0.01 vs control, ^#^*p*<0.05, ^##^*p*<0.01 vs PCS [100 μM], One-way Anova.

Similarly, PCS-treated RMCs showed elevation of phospho-ASK1 level, although not statistically significant (*p* = 0.0906 vs control, unpaired t-test) (Fig 8A, i). ASK1 activation was reduced with pre-treatment of Probenecid and G226 (100 μM and 1.0 μM, respectively). Increased activation of downstream MAPKs (ERK1/2 and p38MAPK) and NF-ĸB were also evident, and were suppressed by Probenecid (100 μM) and G226 (1.0 μM) (Fig 8A, ii-iv).

Additionally, RWJ-67657 and U0126 also suppressed p38MAPK and ERK1/2 activated by PCS, respectively (8B, i-ii). Both inhibitors also significantly abrogated downstream NF-ĸB activation (Fig 8B, iii). Pan Actin remained unchanged in this experimental condition (Fig 8B, iv).

### Indoxyl sulfate and *p*-cresol sulfate increase the gene expression of markers of cardiac hypertrophy and cardiac and renal fibrosis

**Fig 9:**
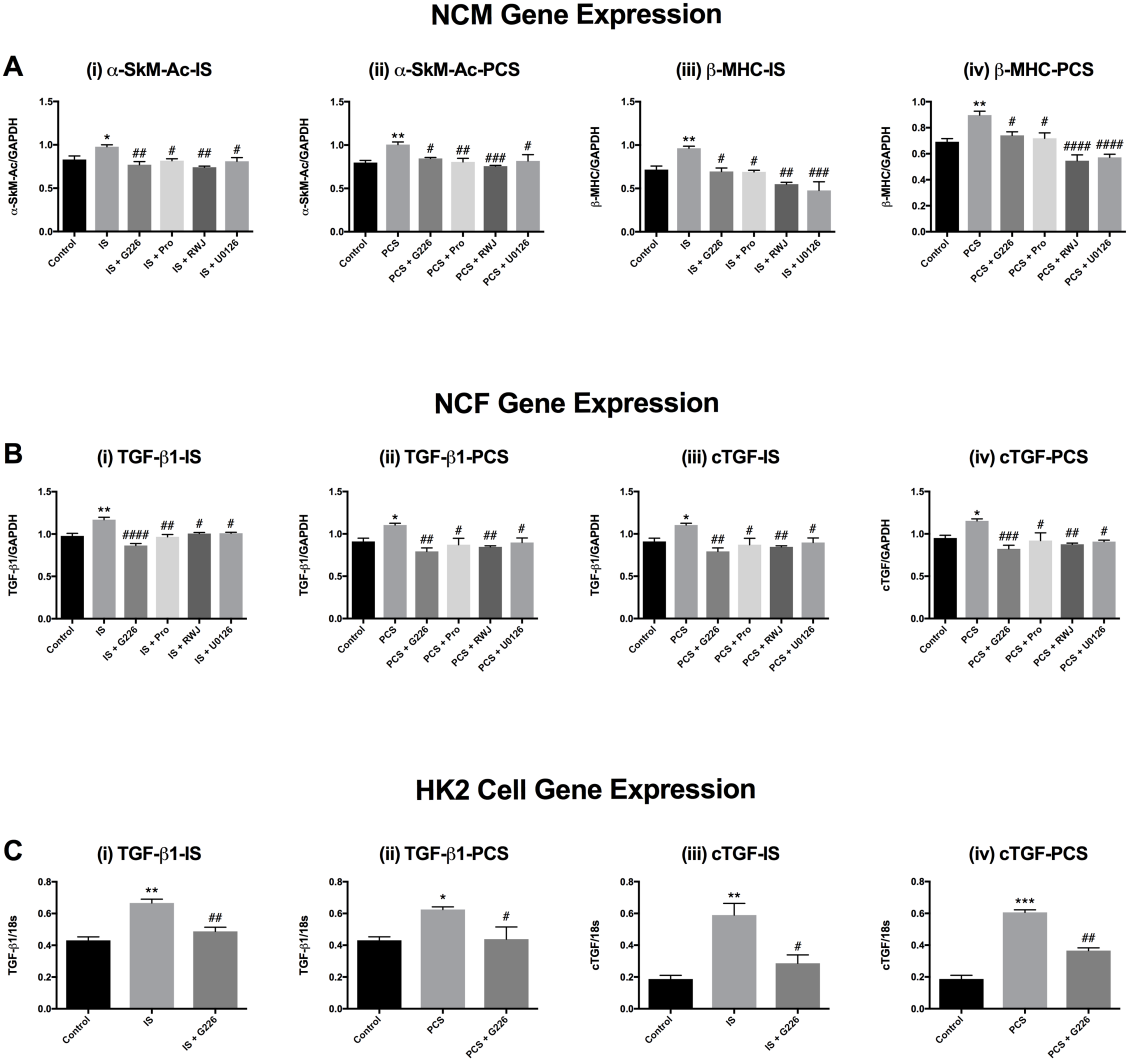
Effects of indoxyl sulfate and *p*-cresol sulfate stimulated on pro-hypertrophic gene expression of NCMs and pro-fibrotic gene expression of NCFs and HK2 cells. IS (10 μM) and PCS (100 μM) upregulated gene expression of α-SkM-Ac and β-MHC in NCMs (A, i-iv) and TGF-β1 and *ctgf* in NCFs (B, i-iv) and HK2 cells (C, i-iv), which were suppressed by G226, Probenecid, RWJ-67657 and U0126. *p<0.05, **p<0.01, ***p<0.001 IS [10 μM] or PCS [100 μM] vs control, ^#^*p*<0.05, ^##^*p*<0.01, ^###^*p*<0.001^####^*p*<0.0001 vs IS [10 μM] or PCS [100 μM], One-way Anova. These results are representatives of two independent experiments in triplicates.

18 hours of incubation with IS (10 μM) and PCS (100 μM) increased gene expression of pro-hypertrophic markers (α-SkM-Ac and β-MHC) in NCMs, as well as pro-fibrotic markers (TGF-β1 and *ctgf*) in NCFs and HK2 cells (Fig 9). G226 (1.0 μM), Pro (100 μM), RWJ-67657 (3.0 μM) and U0126 (1.0 μM) suppressed the expression of α-SkM-Ac and β-MHC in NCMs (Fig 9A) and the expression of TGF-β1 and *ctgf* in NCFs (Fig 9B). In addition, G226 (3.0 μM) also attenuated HK2 cell TGF-β1 and *ctgf* gene expression (Fig 9C).

### Indoxyl sulfate and *p*-cresol sulfate do not affect cardiac and renal cell viability

**Fig 10:**
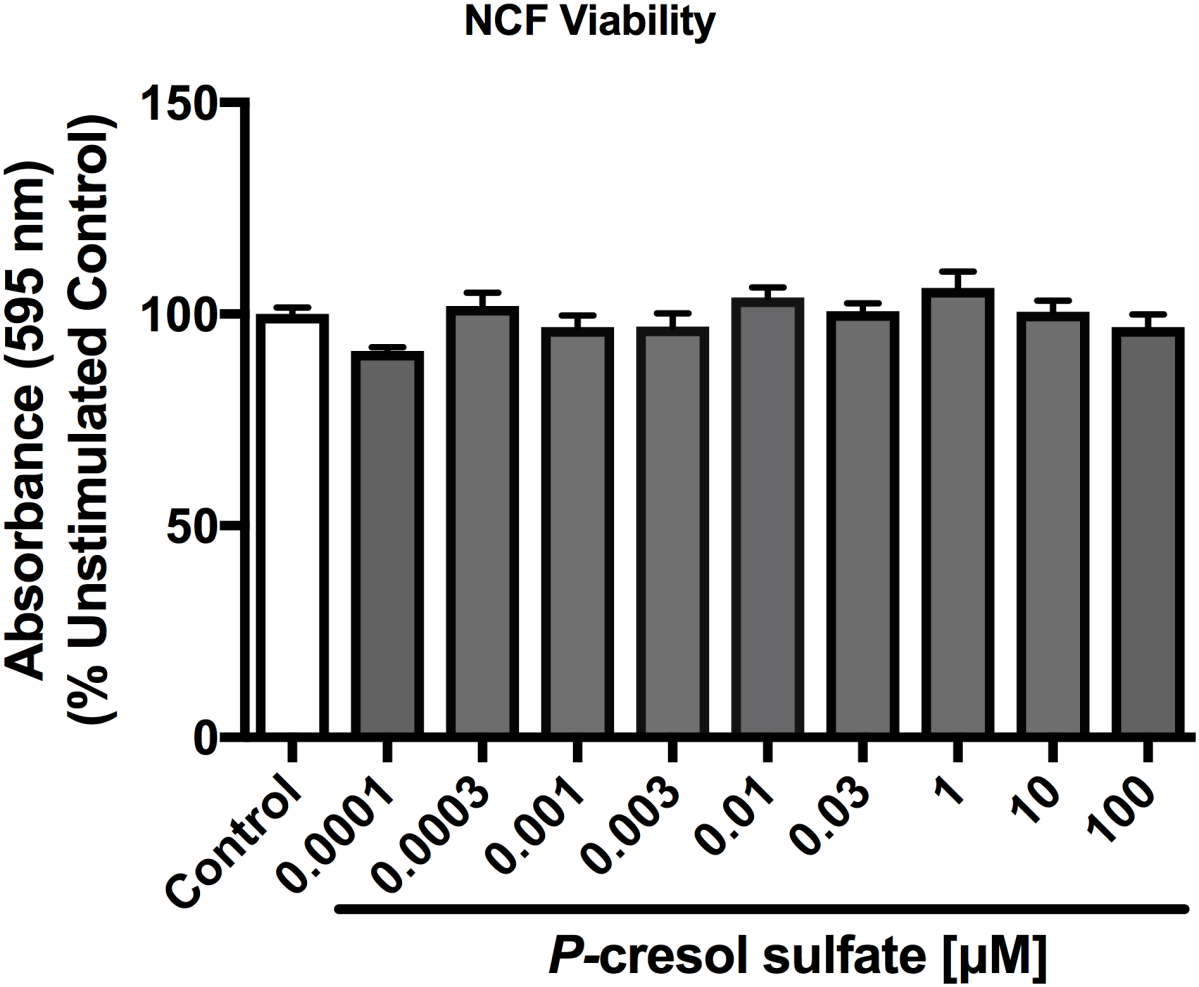
Effects of *p*-cresol sulfate on cardiac cell viability. PCS (0.0001 to 100 μM) did not alter NCF viability as determined by MTT assay. Data are presented as mean ± SEM, each with quadruplets from three independent experiments and analyzed with One-way Anova.

**Fig 11:**
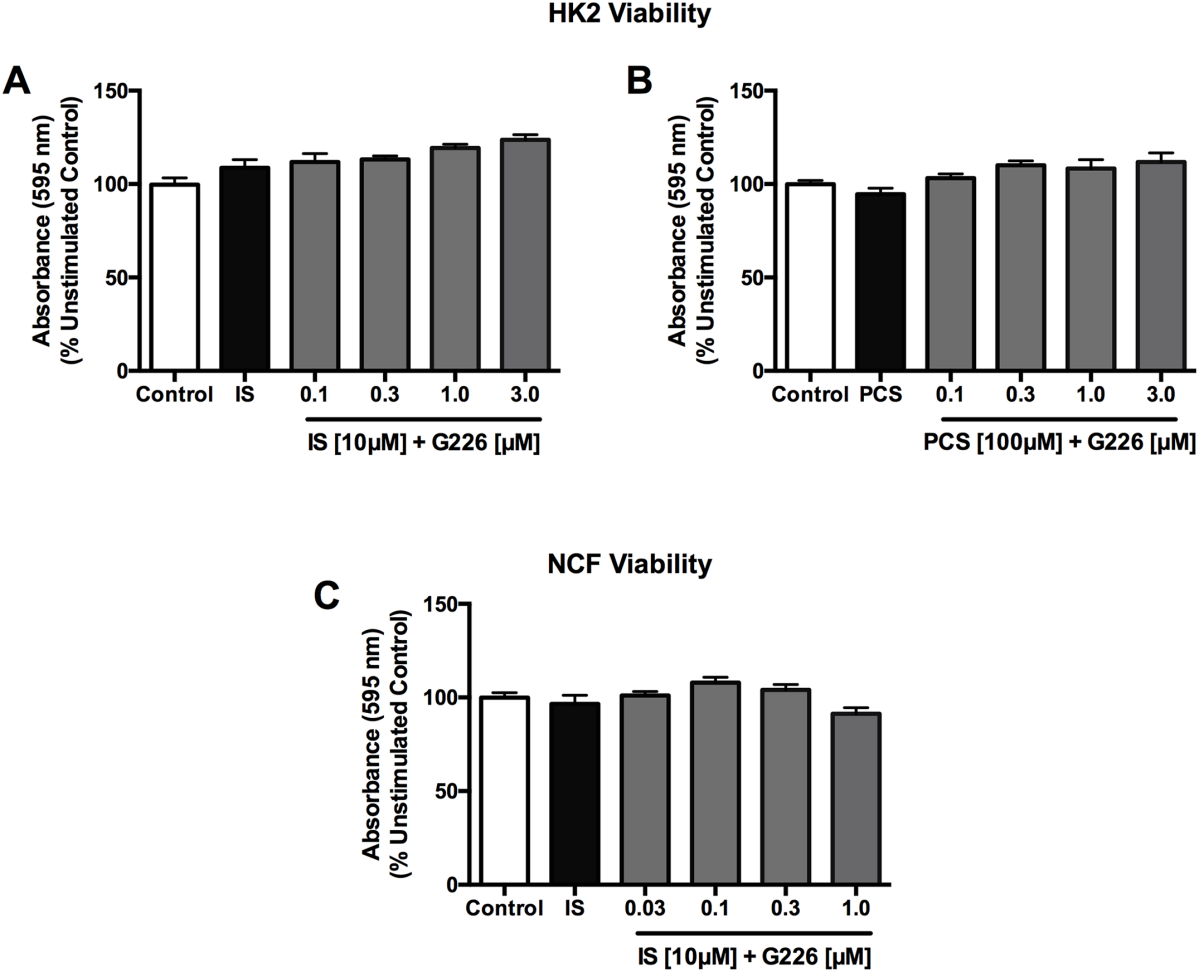
Effects of indoxyl sulfate and *p*-cresol sulfate stimulation with and without selective ASK1 inhibitor on HK2 cell and neonatal cardiac fibroblast viability. MTT assay showed both IS and PCS stimulations (10 and 100 μM, respectively) (A) do not alter cellular viability of HK2 cell and NCFs in the presence and absence of G226 (HK2: 0.1 to 3.0 μM; NCF: 0.03 to 1.0 μM). Data are presented as mean ± SEM of quadruplets from three independent experiments and analyzed with One-way Anova.

3-(4,5-dimethyl-2thiazoyl)-2,5-diphenyl-2H-tetrazolium bromide (MTT) assay indicates that treatment with PCS at concentrations ranging from 0.0001 to 100 μM did not alter viability of NCF (Fig 10). In HK2 cells and NCFs, 48-hour incubation with uremic toxin stimulants (10 μM IS and 100 μM PCS) with or without G226 pre-treatment (2 hours, HK2: 0.1 to 3,0 μM; NCF: 0.03 to 1.0 μM) did not affect the viability of these cells (Fig 11A–11C).

## Discussion

In the present study, we have identified potential direct detrimental effects and mechanistic pathways of protein-bound uremic toxins, IS and PCS, on cardiac and renal cellular functions. ASK1 inhibitor (G226) effectively inhibited cellular remodeling (myocyte hypertrophy and collagen synthesis of cardiac and renal cells) induced by IS and PCS. Mechanistic studies also reveal the activation of ASK1 and downstream MAPKs, ERK1/2 and p38MAPK (both cardiac and renal cells) as well as NFκB (as has been demonstrated with renal cells) pathways as well as the increase in pro-hypertrophic and pro-fibrotic genes by IS and PCS, all of which were suppressed by G226. Overall, we have demonstrated the central role of ASK1 in mediating cardiac hypertrophy and cardiorenal fibrosis elicited by IS and PCS, as well as the inhibitory effect of ASK1 inhibitor in such settings.

IS and PCS are particularly concerning because they are difficult to remove by conventional dialysis due to their substantial protein-binding capacity [9, 11]. More than 90% of IS and PCS are bound to albumin and only 30% are cleared by dialysis, leading to accumulation of these toxins in the serum of CKD patients [9]. Although modest, the increase in hypertrophy and collagen synthesis by IS and PCS shown by leucine and proline incorporation in this study are statistically significant and may be translated into relevant pathophysiological changes *in vivo* as have been demonstrated by other therapies employed with similar protocol [24], therefore clinical relevancy cannot be undermined. Moreover, both IS and PCS increase crucial pro-hypertrophic (α-SkM-Ac and β-MHC) and pro-fibrotic genes (TGF-β1 & *ctgf*). In pathological conditions, the upregulation of these genes signify cellular insults, where cardiac myocyte hypertrophy and cardiac and renal fibrosis occurs as compensatory mechanisms [25–28]. Prolonged cardiac hypertrophy and cardiorenal fibrosis are critical processes in mediating progressive cardiac and renal failure.

Doses of IS and PCS (10 and 100 μM, respectively) tested in this study are clinically relevant, as the pathophysiological concentration of these protein-bound uremic toxins range from a few up to hundreds of micro-molars in the circulation of CKD patients [6, 29]. We have also validated the use of these concentrations in our previous studies with IS [11, 29]. It is important to note that most *in vitro* studies found in the literature utilize high concentration of IS and PCS to mimic end-stage renal disease (mostly above 500 μM), with a huge emphasis on apoptosis/senescence caused by these uremic solutes in various cell types. Our major focus is pro-fibrotic and pro-hypertrophic outcomes in cardiac and renal cells. These cellular processes are observable at much lower concentration (as suggested by our data in this study and in our previous reports [11, 29]), which is more relevant to the pre-dialysis concentration of these solutes [6]. This further underlies the need to shift our attention for the prevention of uremic toxin accumulation or abolishment of their biological effect before renal impairment becomes irreversible.

**Fig 12:**
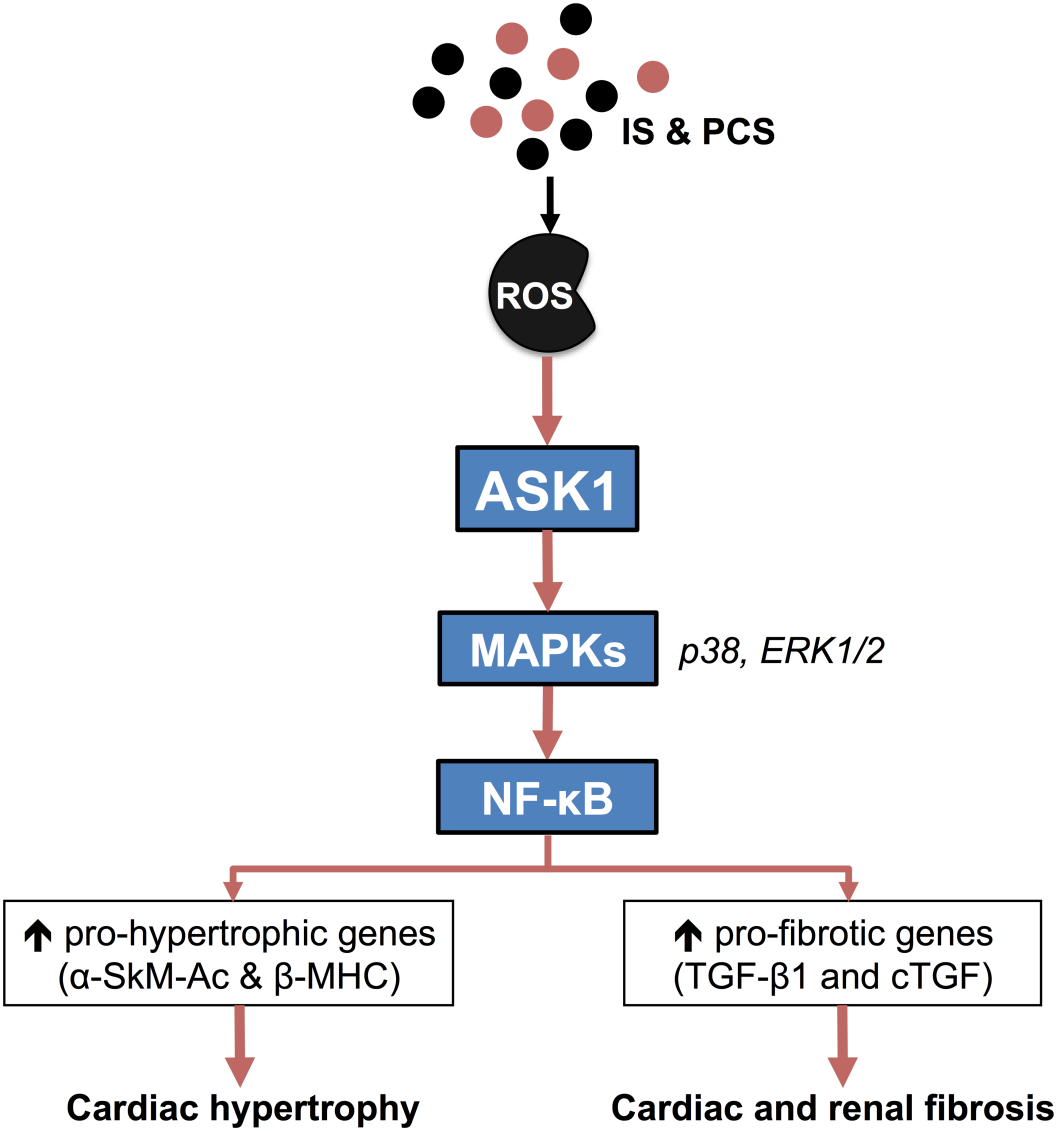
Key findings. While ROS activation by IS and PCS have been widely studied, we have shown in this study that IS and PCS activates ASK1, a ROS-driven protein kinase, and its downstream MAPKs (p38MAPK and ERK1/2) as well as NF-κB, leading to the upregulation of fetal genes (α-SkM-Ac and β-MHC) to promote cardiac hypertrophy and pro-fibrotic genes (TGF-β1 and *ctgf*) to cause cardiac and renal fibrosis.

We have previously demonstrated direct pro-hypertrophic and pro-fibrotic effects of IS on cardiac myocytes and fibroblasts, respectively [11]. In this study, we have further established a key oxidative stress-signaling pathway, ASK1, to be involved in IS-mediated hypertrophy and collagen synthesis on cardiac cells. This was further supported with apparent increase in gene expression of and β-MHC (pro-hypertrophic markers) as well as *ctgf* and TGF-β1 (pro-fibrotic markers). Previous studies have suggested IS initiates cardiac fibrosis *via* ROS-NF-κB-TGF-β1 pathway [30]. The uptake of IS by OATs has also been localized to the tubular cells of subtotal nephrectomized (STNx) rats [19]–and blockade of OAT1/3 by Probenecid diminished detrimental effects of IS on cardiac cells [29]. We have also determined the activation of MAPKs (p38MAPK and ERK1/2) and NF-κB in IS-induced cardiac cellular remodeling [11]. Therefore, in pathological settings, the toxic effects caused by IS could involve its entry of the target cell *via* OAT1/3 followed by the activation of ROS-ASK1-MAPKs (p38/ERK1/2)-NF-κB cascade, leading to increased expression of pro-hypertrophic (α-SkM-Ac and β-MHC) and pro-fibrotic genes (TGF-β1 & *ctgf*) and ultimately resulting in cardiac hypertrophy and fibrosis (Fig 12).

The effect of PCS on cardiac cellular functions remains poorly understood. In addition to the pro-apoptotic effect on cardiac myocytes as a previous study suggests [31], our results indicate PCS additionally possess pro-hypertrophic and pro-fibrotic attributes that may directly mediate its adverse effects on cardiac cellular functions *via* similar mechanisms as described above for IS (Fig 12). Additionally, noting that OAT1/3 is a high capacity transporter for PCS [20] just as it is for IS, Probenecid (OAT1/3 inhibitor) attenuated PCS-induced cardiac cellular hypertrophy and collagen synthesis. Although the inhibition by OAT1/3 inhibitor is evident, the active transport of IS and PCS by OAT1/3 in heart cells remains to be proven. Furthermore, the phosphorylation of NF-κB was not significantly elevated with PCS stimulation in NCM at the time point of the assay performed, although it was activated by IS at similar time point as we have shown previously [11]. This could mean that PCS mediates inflammatory effects *via* other pro-inflammatory molecules, or the time point of our assay missed the detection of phospho-NF-κB—further investigation is needed.

Renal oxidative stress associated with protein-bound metabolites have been extensively studied, especially related to IS and PCS [7, 32, 33]. Studies have shown that ROS-NF-κB-TGF-β1 pathways are implicated in the IS- and PCS-associated dysfunction of mesangial and tubular cells, possibly leading to renal fibrosis—a mechanism similar to that of IS-induced cardiac fibrosis [32]. In this study, we have shown for the first time, IS and PCS have direct and deleterious pro-fibrotic effects on renal cellular remodeling process. Both uremic toxins increased RMC and HK2 cell collagen turnover after 48 hours of incubation, possibly mediated by ASK1 and its downstream MAPKs (p38MAPK and ERK1/2), followed by subsequent upregulation of TGF-β1 and *ctgf* gene expression. Although involvements of MAPKs have been demonstrated in previous studies [34, 35], this is the first time ASK1 activation is unveiled under the influence of IS and PCS in renal cells. NF-κB pathway was also activated; indicating IS- and PCS-initiated kidney fibrosis may also entail an inflammatory reaction *via* NF-κB phosphorylation. This is in concordant with a previous study where free radical-initiated activation of NF-κB by IS was observed in renal tubular and mesangial cells [36]. Overall, IS- and PCS-mediated renal cellular fibrosis entails identical signaling cascade as observed in cardiac fibroblasts (Fig 12).

It is of note the observations discussed above occurred without alteration to the viability of both cardiac and renal cells tested as demonstrated by MTT assay. This strongly suggests the inhibitory effect exhibited by the inhibitors used was due to actual abrogation of targeted ASK1 and other intended pathways. Also, the range of IS and PCS concentration used in this study are within the capability of the cells to stay viable in our experimental conditions for intended observations (hypertrophy and collagen synthesis) to be made.

There are ways to directly address uremic toxin accumulation. AST-120 is a well-studied IS adsorbent that has shown beneficial outcome in ameliorating cardiac dysfunction *in vivo* [37]. Antagonism of OAT1/3 have also displayed beneficial outcome in attenuating the uptake of IS and PCS [20, 29], which we have also shown in this study. However, these treatments are ineffective against uremic toxins that have already accumulated within cells. AST-120 only binds to the precursor of IS in the intestinal tracts, while OAT1/3 antagonists only blocks IS and PCS uptake on the cellular membrane. Targeting intracellular pathway activated by these toxic solutes may be more useful as an adjunctive form of therapy with current therapeutic regimen for CRS, which is mainly empirical [38].

There are some limitations to this study. First, cardiac cells cultured from neonatal rats may have different metabolism [39] and electrophysiological characteristics [40] as opposed to cardiac cells from adult rats. However, they are cost-effective and much easier to grow and maintain [39] to evaluate cellular functions such as hypertrophy and fibrosis (determined by the level of collagen synthesis) and for assessment of drug efficacy *in vitro* [11]. Cardiac cells from rat neonates also possess identical phenotype and express similar early fetal genes (e.g. β-MHC) as adult cardiac cells under pathological conditions [11, 41–43]. Similarly, established renal cell lines are commonly used to examine basic renal physiology and pathology such as collagen synthesis and drug potency at the cellular level [44]. Secondly, the complexity of CRS cannot be represented by any kind of *in vitro* model. Despite that, *in vitro* model provides the ability to control minimal, yet highly specific conditions for any experiments—in this case, for the evaluation of the direct effects of uremic toxins on cardiac and renal cells, with subpar impeding factors. Additionally, although beneficial inhibitory effects have been observed in this study, further *in vivo* and clinical evaluations using the ASK1 inhibitor are warranted. Currently, there is no effective ASK1 inhibitor for the treatment of cardiac and renal dysfunctions available, whereby lack of efficacy and high cellular toxicity are some of the common issues faced. We sought to rationalize the ASK1 inhibitor, G226, to be considered for further assessment, as *in vitro* studies enable direct evaluation of drug efficacy without interference by other systems such as in an *in vivo* and clinical setting [29]. The results of our studies, as a proof of concept, suggest ASK1 inhibitor alone may be sufficient to hinder over-activation of ASK1 and downstream MAPKs (at least ERK1/2 and p38MAPK) and NF-κB by IS and PCS.

In summary, IS and PCS enhance expression of pro-fibrotic genes and increase levels of proteins related to hypertrophy and fibrosis *in vitro*, at least in part *via* the activation of ASK1 and other downstream signaling pathways i.e. p38MAPK, ERK1/2 and NF-κB through the OAT1/3 channels. ASK1 inhibitor attenuated cardiac hypertrophy and cardiorenal fibrosis induced by IS and PCS, and may be a beneficial therapeutic agent to decelerate CRS progression mediated by uremic toxins.

## Supporting information

S1 Table List of antibodies for Western blot analysis. (PDF)

S2 Table List of primer pairs sequence for real-time PCR. (PDF)

S1 Fig Original Western blot membrane of NCMs stimulated with PCS and treated with Probenecid and ASK1 inhibitor. (A) phospho-ASK1, (B) pan-actin, (C) phospho-p38, (D) total p38, (E) phospho-ERK1/2, (F) total ERK1/2, (G) phospho-NF-κB, (H) total NF-κB. Order in triplicates: Control, PCS, PCS+Pro, PCS+G226.
(PDF)

S2 Fig Original Western blot membrane of NCMs stimulated with PCS and treated with RWJ-67657 and U0126. (A) phospho-p38, (B) total p38, (C) phospho-ERK1/2, (D) total ERK1/2, (E) phospho-NF-κB, (F) total NF-κB, (G) pan-actin. Order in triplicates: Control, PCS, PCS+RWJ, PCS+U0126.
(PDF)

S3 Fig Original Western blot membrane of RMCs stimulated with IS and treated with Probenecid and ASK1 inhibitor. (A) phospho-ASK1, (B) pan-actin, (C) phospho-p38, (D) total p38, (E) phospho-ERK1/2, (F) total ERK1/2, (G) phospho-NF-κB, (H) total NF-κB. Order in triplicates: Control, IS, IS+Pro, IS+G226.
(PDF)

S4 Fig Original Western blot membrane of RMCs stimulated with IS and treated with RWJ-67657 and U0126. (A) phospho-p38, (B) total p38, (C) phospho-ERK1/2, (D) total ERK1/2, (E) phospho-NF-κB, (F) total NF-κB, (G) pan-actin. Order in triplicates: Control, IS, IS+RWJ, IS+U0126.
(PDF)

S5 Fig Original Western blot membrane of RMCs stimulated with PCS and treated with Probenecid and ASK1 inhibitor. (A) phospho-ASK1, (B) pan-actin, (C) phospho-p38, (D) total p38, (E) phospho-ERK1/2, (F) total ERK1/2, (G) phospho-NF-κB, (H) total NF-κB. Order in triplicates: Control, PCS, PCS+Pro, PCS+G226.
(PDF)

S6 Fig Original Western blot membrane of RMCs stimulated with PCS and treated with RWJ-67657 and U0126. (A) phospho-p38, (B) total p38, (C) phospho-ERK1/2, (D) total ERK1/2, (E) phospho-NF-κB, (F) total NF-κB, (G) pan-actin. Order in triplicates: Control, PCS, PCS+RWJ, PCS+U0126.
(PDF)
