# Supplementary material for: Apoptosis signal-regulating kinase 1 inhibition attenuates cardiac hypertrophy and cardiorenal fibrosis induced by uremic toxins: Implications for cardiorenal syndrome
Source: PLoS One. 2017 Nov 6;12(11):e0187459. doi: 10.1371/journal.pone.0187459 (PMC5673193; doi:10.1371/journal.pone.0187459)
Supplement: S1 Table — (PDF) [file pone.0187459.s001.pdf]

Primary antibody	Isotype	Dilution	Secondary antibody	Dilution
Anti-phospho-ASK1 (pSer966) SAB4504337 (Sigma)	Rabbit monoclonal IgG	1:1000 in 5% BSA	Anti-rabbit IgG HRP-linked #7074 (Cell Signaling Technology)	1:2000
Phospho-p44/42 MAPK (ERK1/2) #4377S (Cell Signaling Technology)	Rabbit monoclonal IgG	1:4000 in 5% BSA	Anti-rabbit IgG HRP-linked #7074 (Cell Signaling Technology)	1:2000
Phospho-p38 MAPK (T202) #9215L (Cell Signaling Technology)	Rabbit monoclonal IgG	1:2000 in 5% BSA	Anti-rabbit IgG HRP-linked #7074 (Cell Signaling Technology)	1:2000
Phospho-NF-kappaB p65 #3033L (Cell Signaling Technology)	Rabbit monoclonal IgG	1:2000 in 5% BSA	Anti-rabbit IgG HRP-linked #7074 (Cell Signaling Technology)	1:2000
p44/42 MAPK (ERK1/2) #4695S (Cell Signaling Technology)	Rabbit monoclonal IgG	1:4000 in 5% BSA	Anti-rabbit IgG HRP-linked #7074 (Cell Signaling Technology)	1:2000
p38 MAPK #9212 (Cell Signaling Technology)	Rabbit monoclonal IgG	1:2000 in 5% BSA	Anti-rabbit IgG HRP-linked #7074 (Cell Signaling Technology)	1:2000
NF-kappaB p65 #4764S (Cell Signaling Technology)	Rabbit monoclonal IgG	1:2000 in 5% BSA	Anti-rabbit IgG HRP-linked #7074 (Cell Signaling Technology)	1:2000
Pan-actin #ACTN 05 (Neomarkers)	Rabbit monoclonal IgG	1:2000 in 5% blotto	Anti-rabbit IgG HRP-linked #7074 (Cell Signaling Technology)	1:2000
