# Supplementary material for: Apoptosis signal-regulating kinase 1 inhibition attenuates cardiac hypertrophy and cardiorenal fibrosis induced by uremic toxins: Implications for cardiorenal syndrome
Source: PLoS One. 2017 Nov 6;12(11):e0187459. doi: 10.1371/journal.pone.0187459 (PMC5673193; doi:10.1371/journal.pone.0187459)
Supplement: S2 Table — (PDF) [file pone.0187459.s002.pdf]

Gene	Sequence
GAPDH (rat)	For: 5’-GAC ATG CCG CCT GGA GAA AC-3’
	Rev: 5’-AGC CCA GGA TGC CCT TTA GT-3’
18S rRNA	For: 5’-TCG AGG CCC TGT AAT TGG AA-3’
	Rev: 5’-CCC TCC AAT GGA TCC TCG TT-3’
α -SkM-Ac (rat)	For: 5’-TCG CGA CCT TAC TGA CTA CCT G-3’
	Rev: 5’-GCT TCT CTT TGA TGT CGC GC-3’
β-MHC (rat)	For: 5’-TTG GCA CGG ACT GCG TCA TC-3’
	Rev: 5’-GAG CCT CCA GAG TTT GCT GAA GGA-3’
TGF-β1 (rat)	For: 5’-CCA GCC GCG GGA CTC T-3’
	Rev: 5’-TTC CGT TTC ACC AGC TCC AT-3’
cTGF (rat)	For: 5’-GCG GCG AGT CT TCC AA-3’
	Rev: 5’-CCA CGG CCC CAT CCA-3’
TGF-β1 (human)	For: 5’-CCC TGG ACA CCA ACT ATT GC-3’
	Rev: 5’-CCT CCA GCC GAG GTC CTT-‘3
cTGF (human)	For: 5’-GAG GAA AAC ATT AAG AAG GGC AAA-‘3
	Rev: 5’-CGG CAC AGG TCT TGA TGA-‘3
