# Supplementary figures and images for: Apoptosis signal-regulating kinase 1 inhibition attenuates cardiac hypertrophy and cardiorenal fibrosis induced by uremic toxins: Implications for cardiorenal syndrome
Source: PLoS One. 2017 Nov 6;12(11):e0187459. doi: 10.1371/journal.pone.0187459 (PMC5673193; doi:10.1371/journal.pone.0187459)

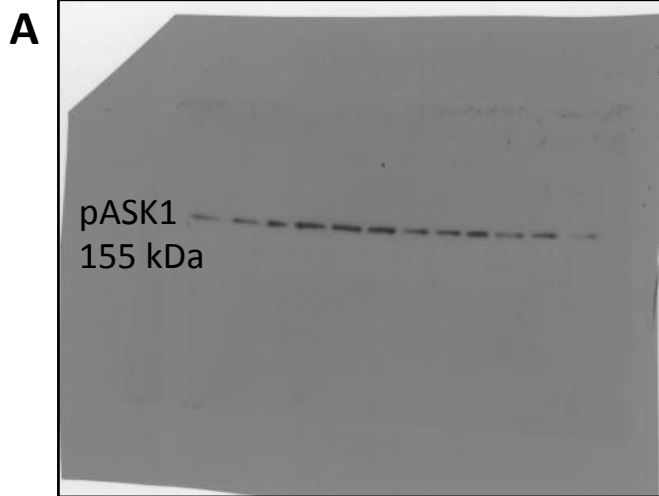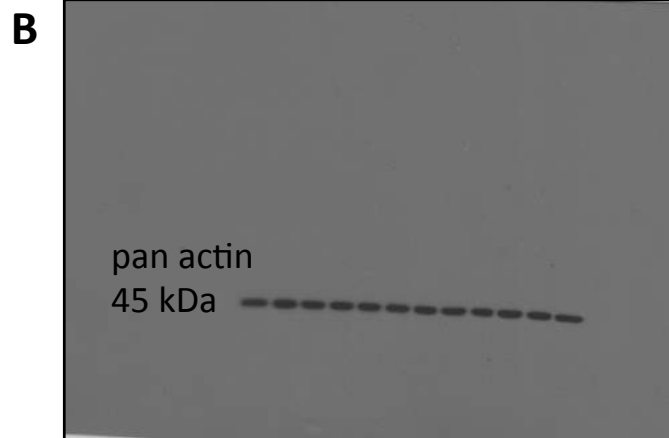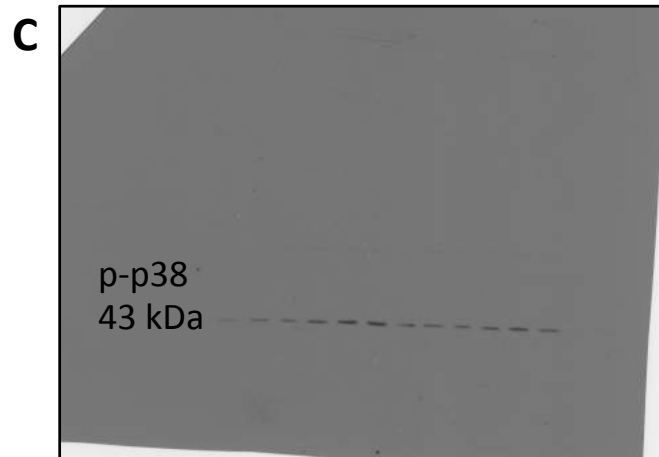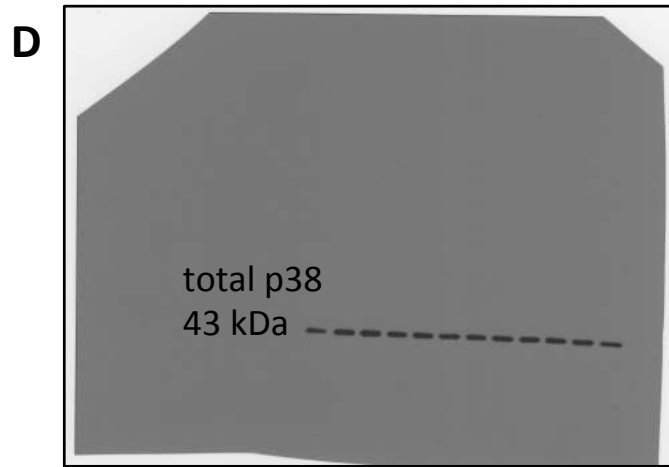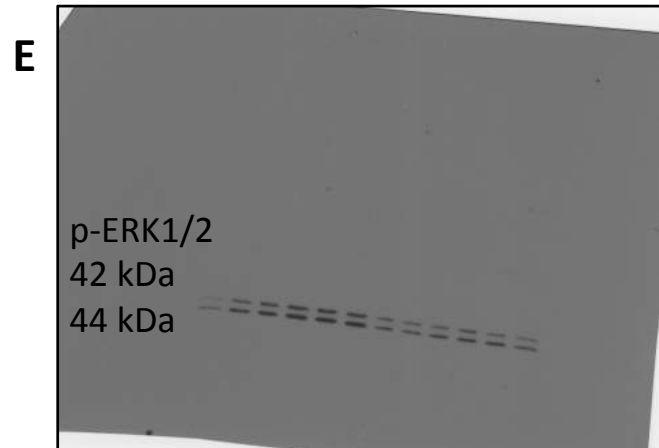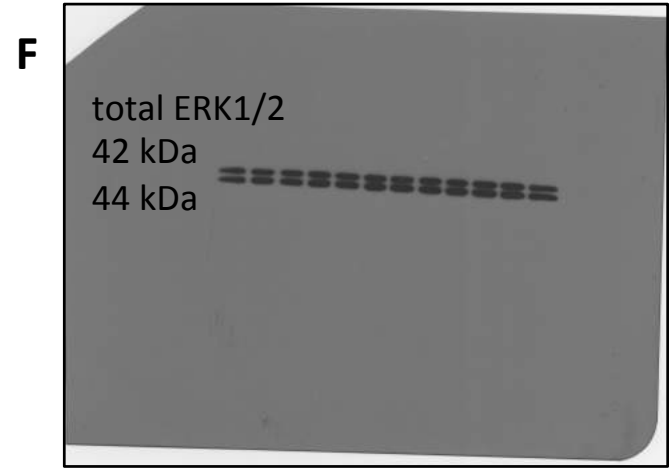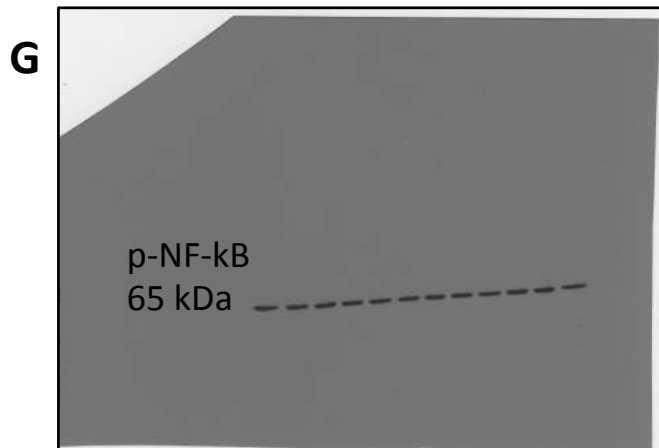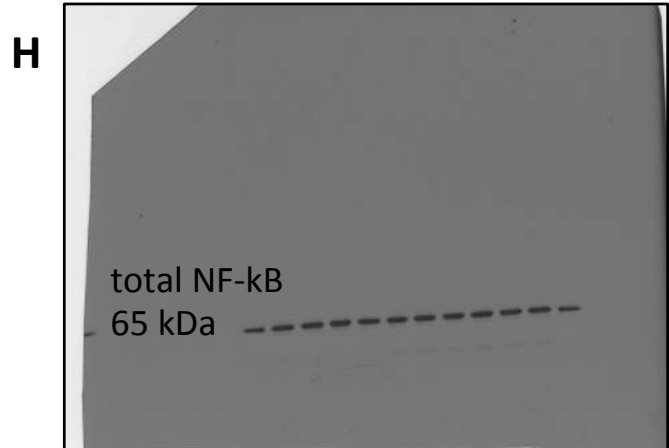

Supplement: S1 Fig — (A) phospho-ASK1, (B) pan-actin, (C) phospho-p38, (D) total p38, (E) phospho-ERK1/2, (F) total ERK1/2, (G) phospho-NF-κB, (H) total NF-κB. Order in triplicates: Control, PCS, PCS+Pro, PCS+G226. (PDF) [file pone.0187459.s003.pdf]

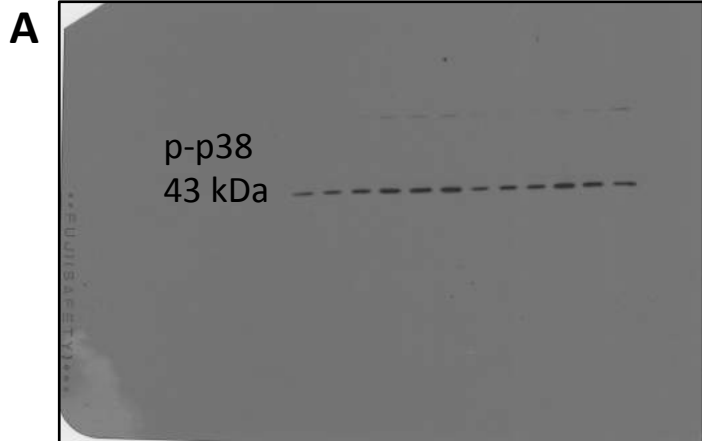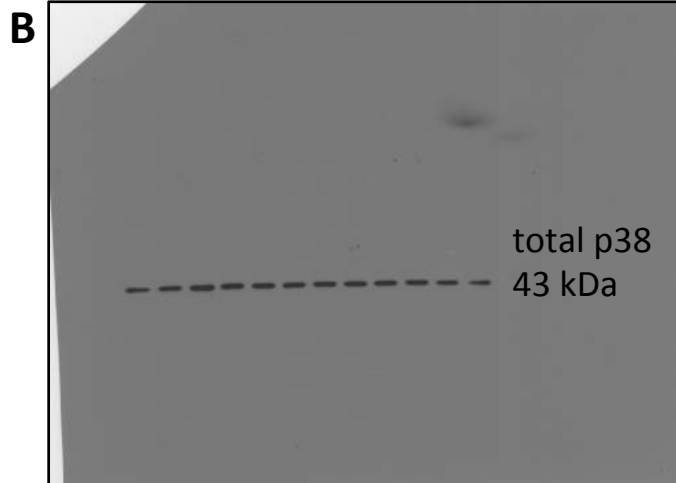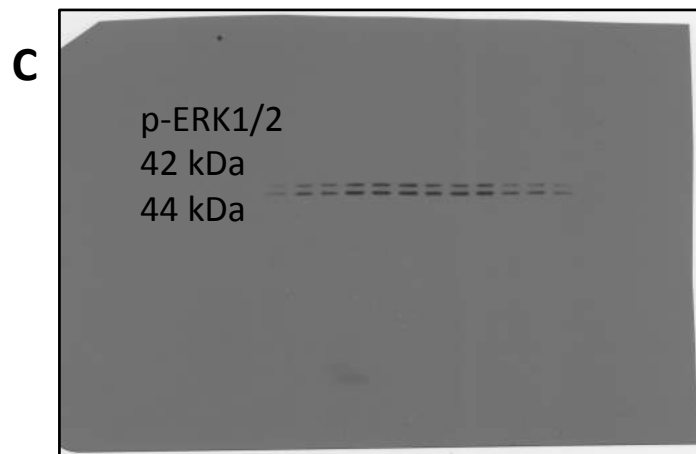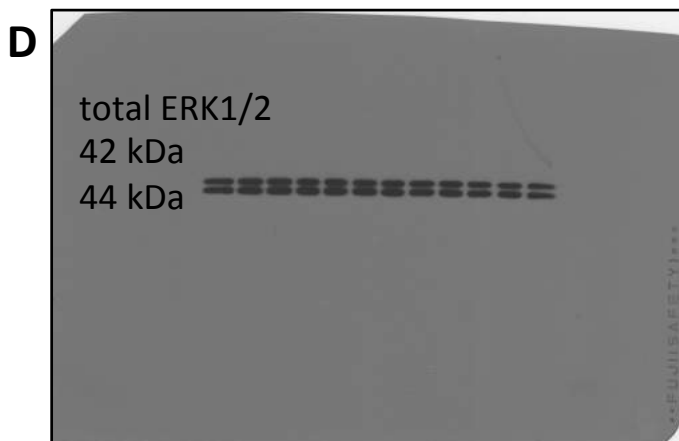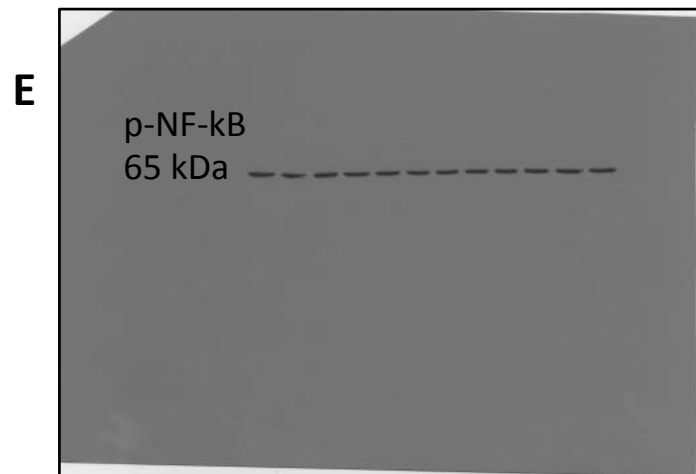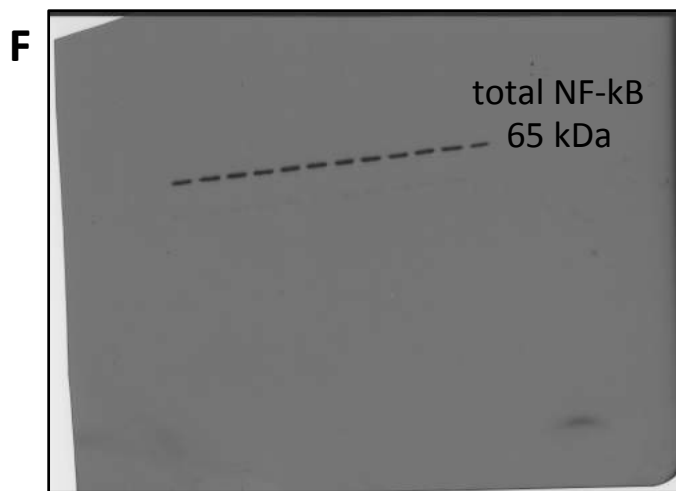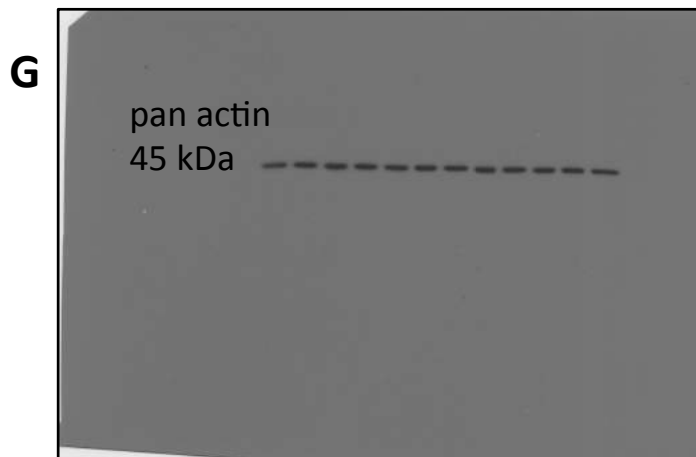

Supplement: S2 Fig — (A) phospho-p38, (B) total p38, (C) phospho-ERK1/2, (D) total ERK1/2, (E) phospho-NF-κB, (F) total NF-κB, (G) pan-actin. Order in triplicates: Control, PCS, PCS+RWJ, PCS+U0126. (PDF) [file pone.0187459.s004.pdf]

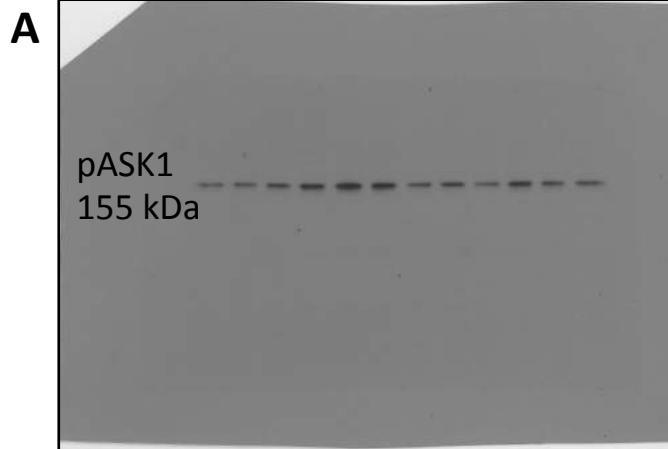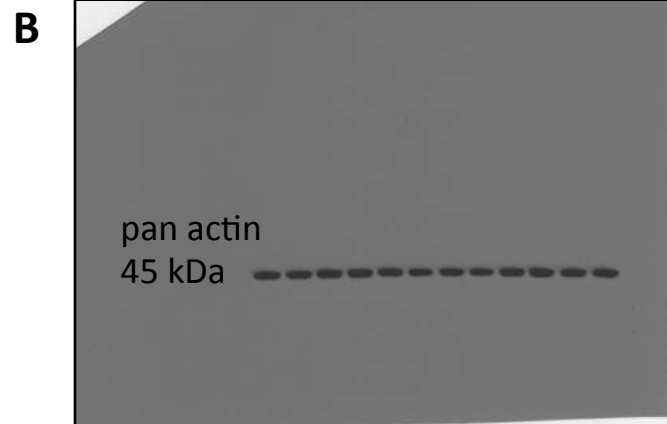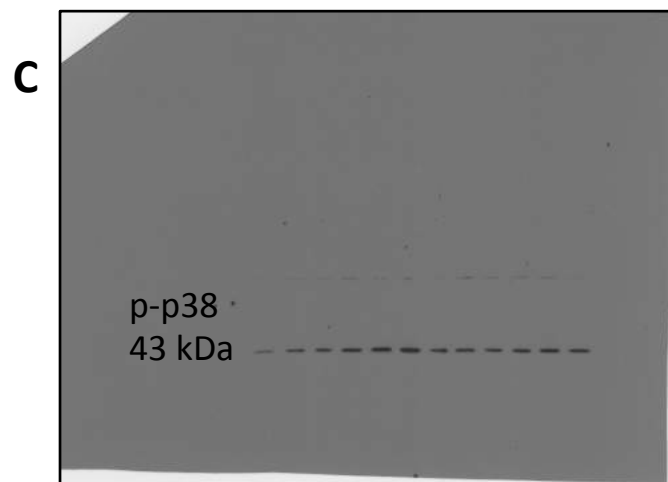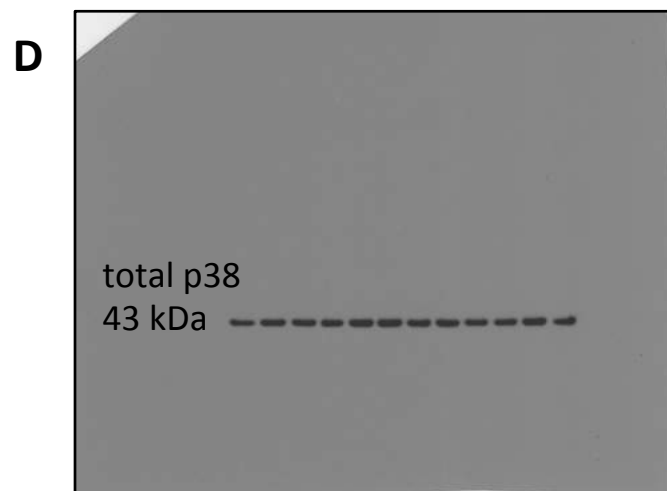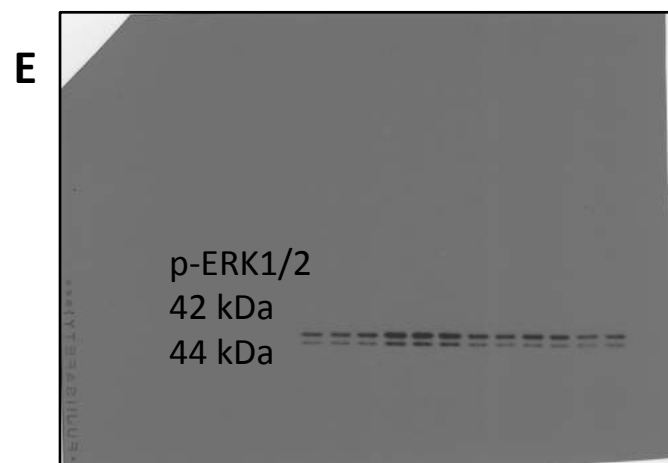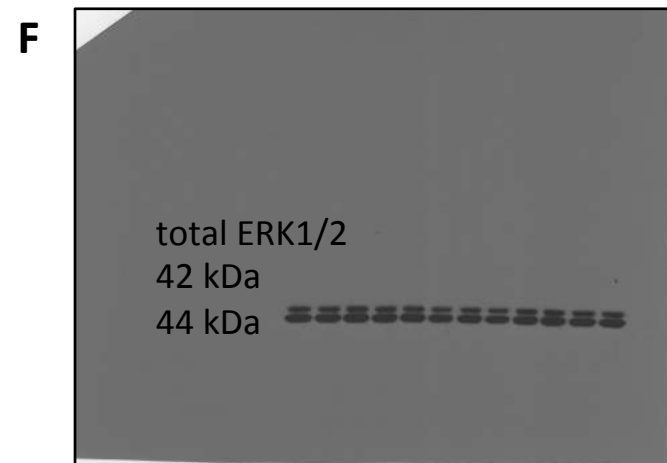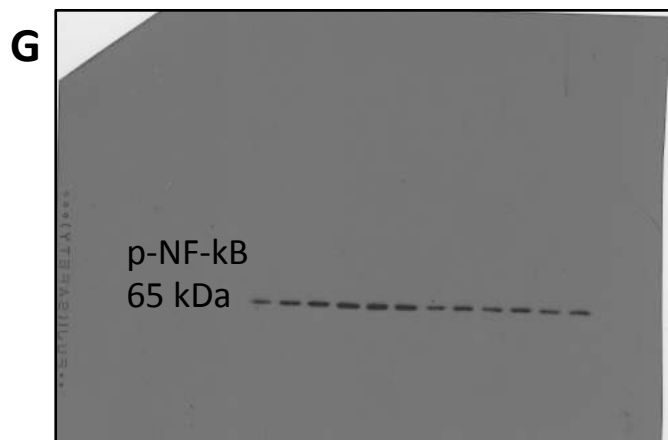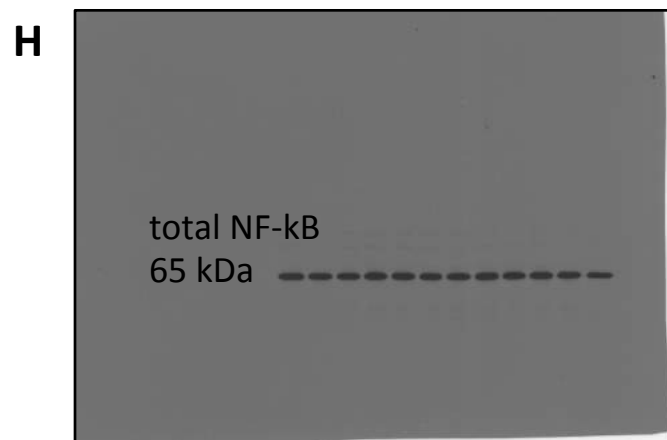

Supplement: S3 Fig — (A) phospho-ASK1, (B) pan-actin, (C) phospho-p38, (D) total p38, (E) phospho-ERK1/2, (F) total ERK1/2, (G) phospho-NF-κB, (H) total NF-κB. Order in triplicates: Control, IS, IS+Pro, IS+G226. (PDF) [file pone.0187459.s005.pdf]

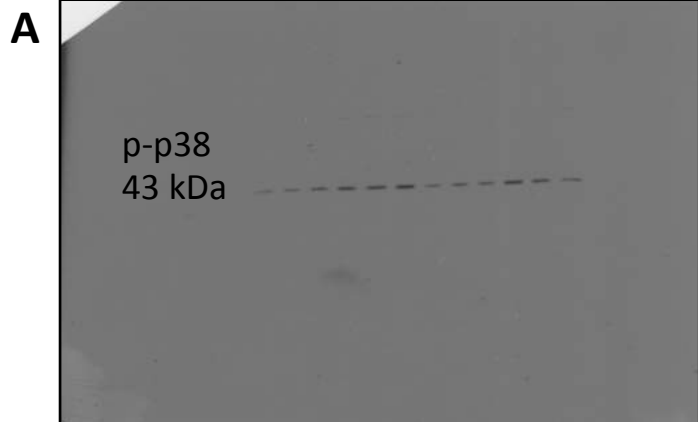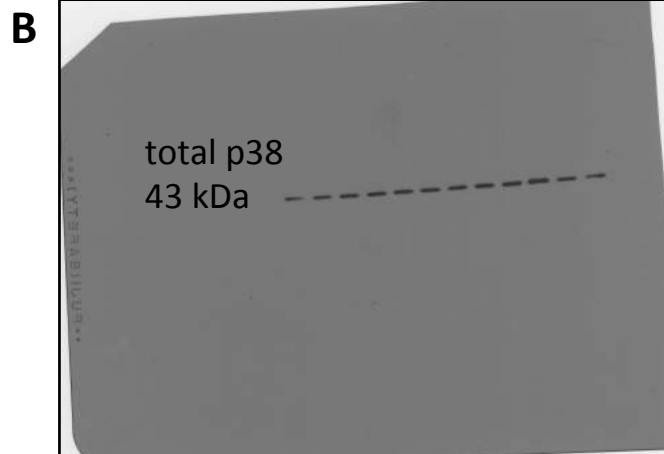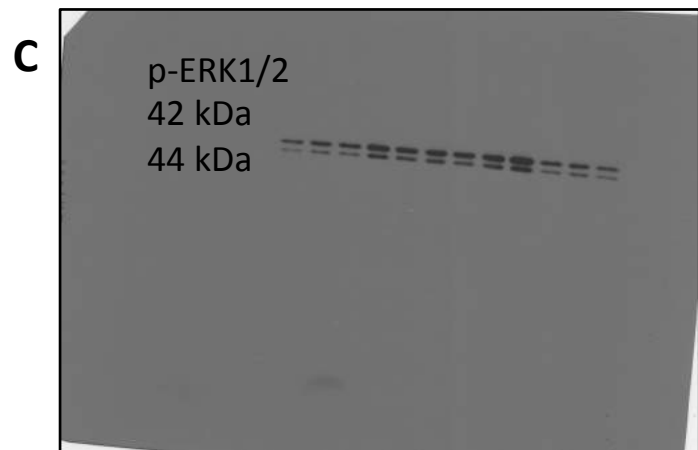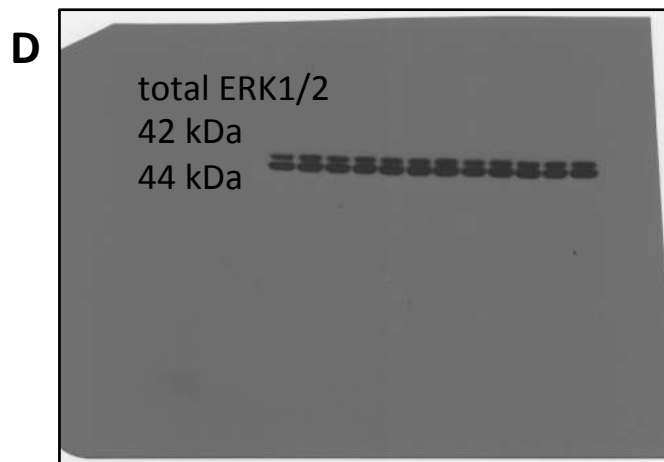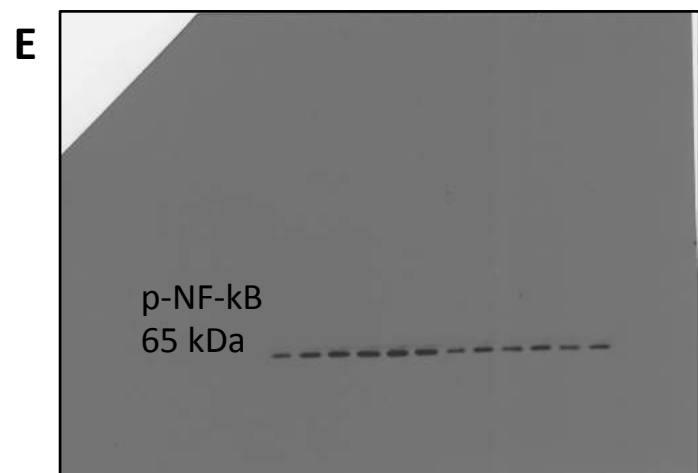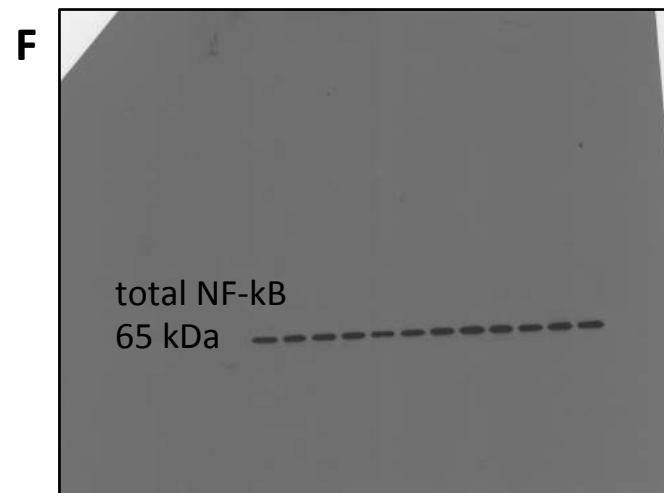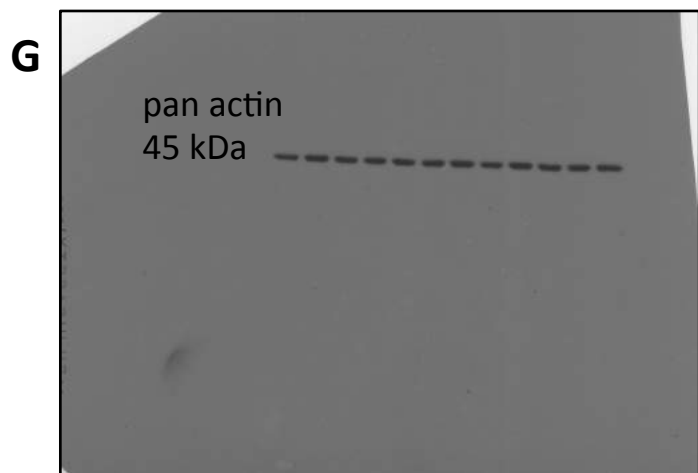

Supplement: S4 Fig — (A) phospho-p38, (B) total p38, (C) phospho-ERK1/2, (D) total ERK1/2, (E) phospho-NF-κB, (F) total NF-κB, (G) pan-actin. Order in triplicates: Control, IS, IS+RWJ, IS+U0126. (PDF) [file pone.0187459.s006.pdf]

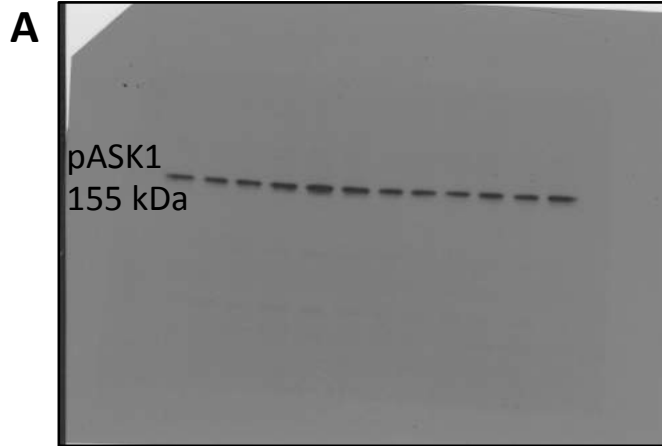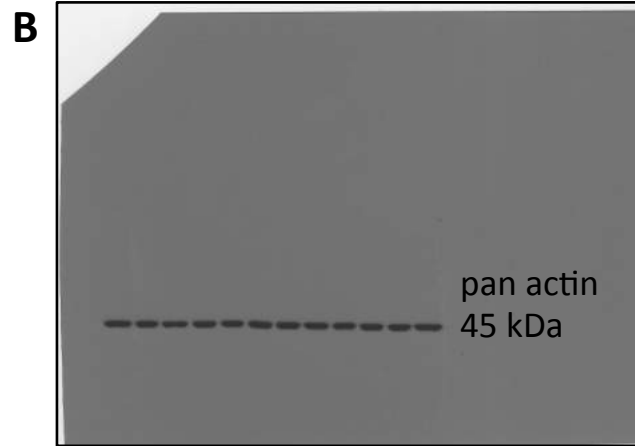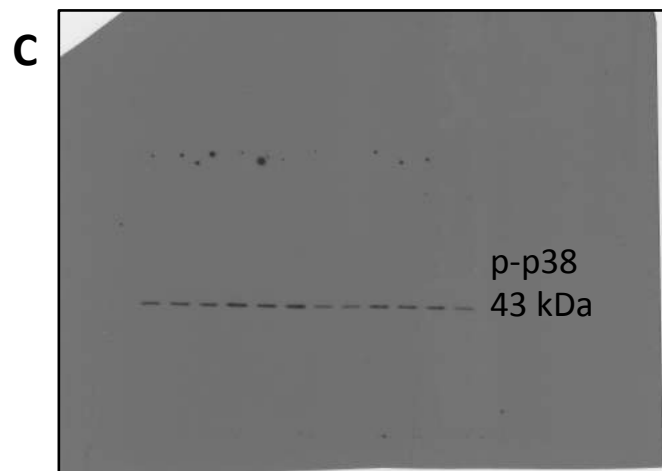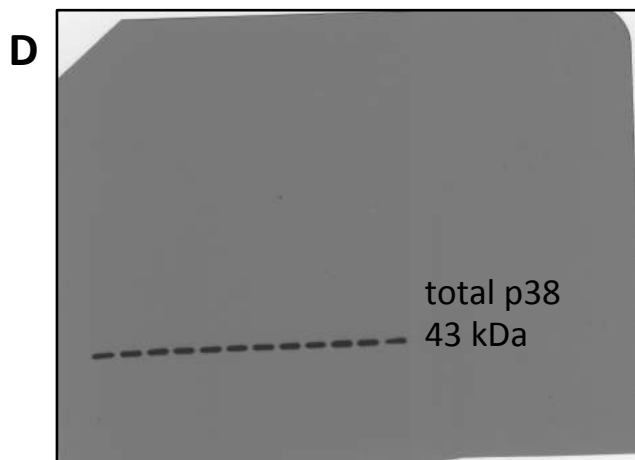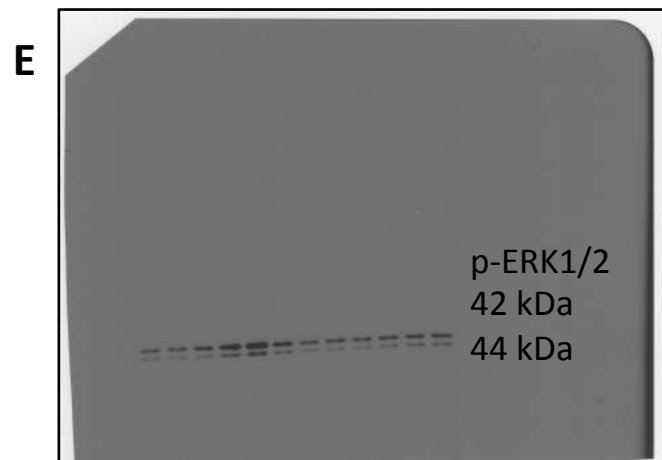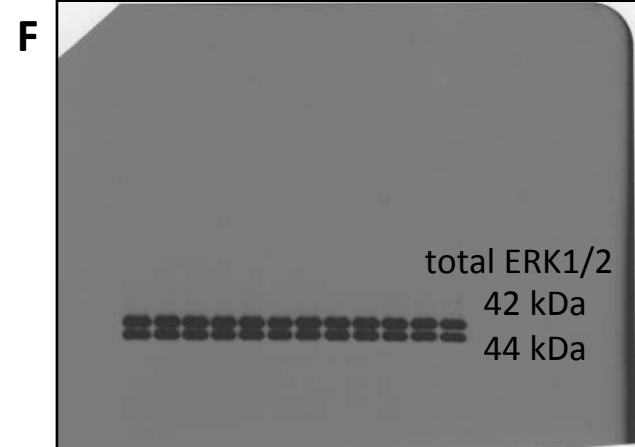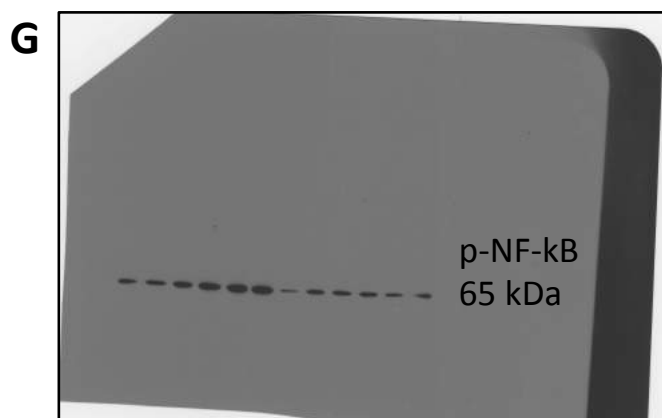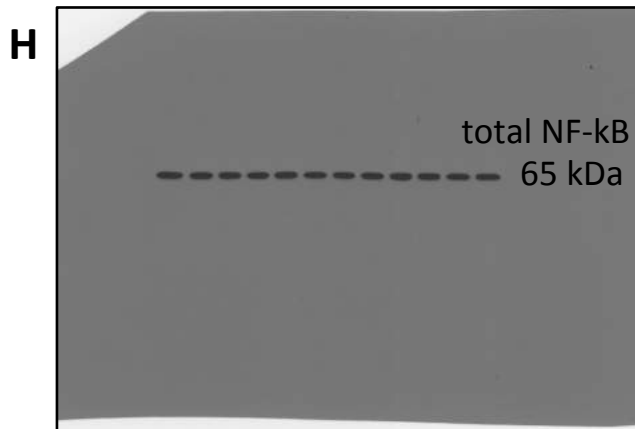

Supplement: S5 Fig — (A) phospho-ASK1, (B) pan-actin, (C) phospho-p38, (D) total p38, (E) phospho-ERK1/2, (F) total ERK1/2, (G) phospho-NF-κB, (H) total NF-κB. Order in triplicates: Control, PCS, PCS+Pro, PCS+G226. (PDF) [file pone.0187459.s007.pdf]

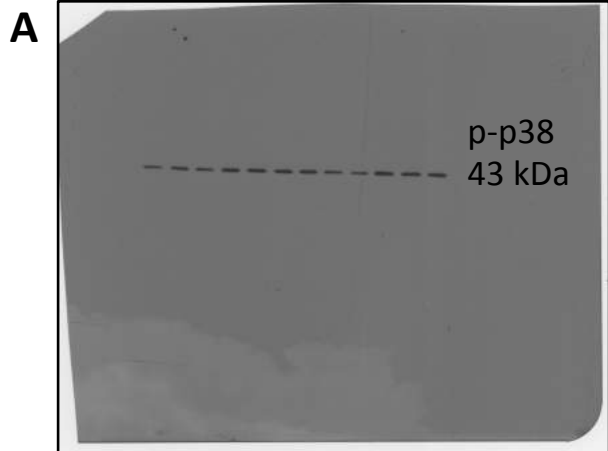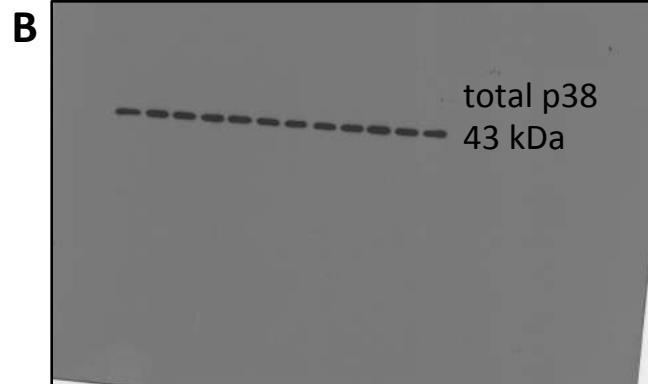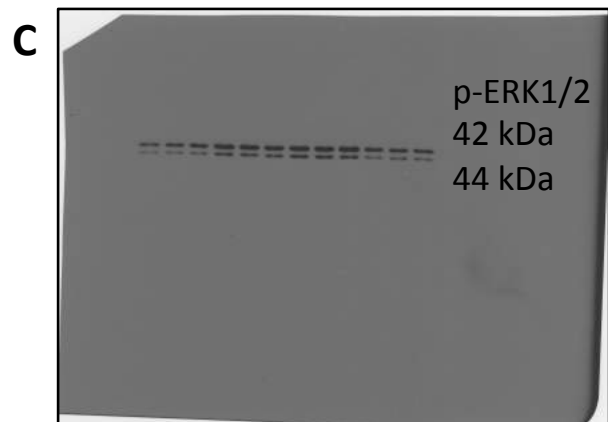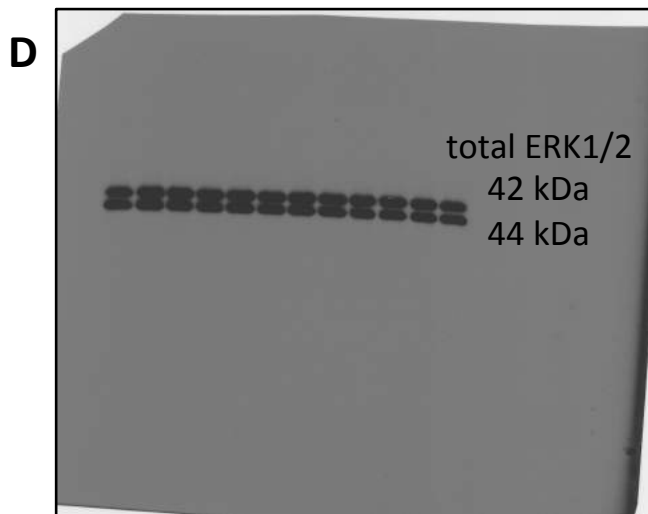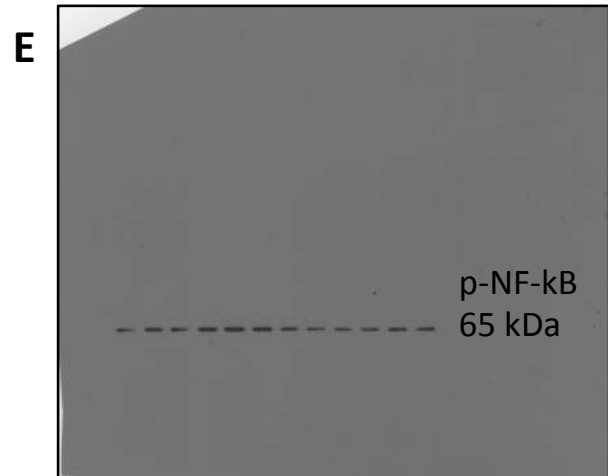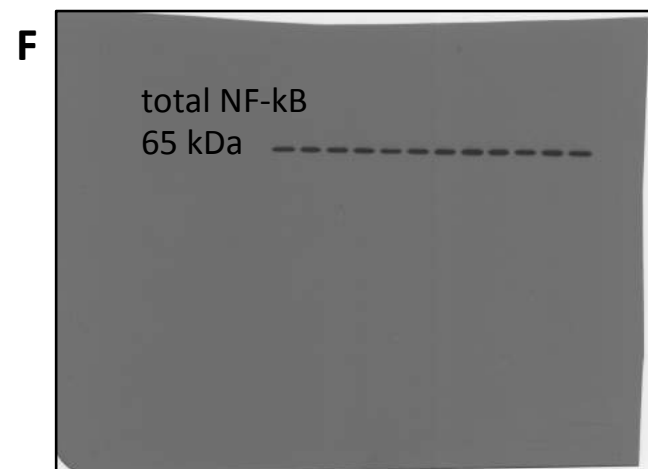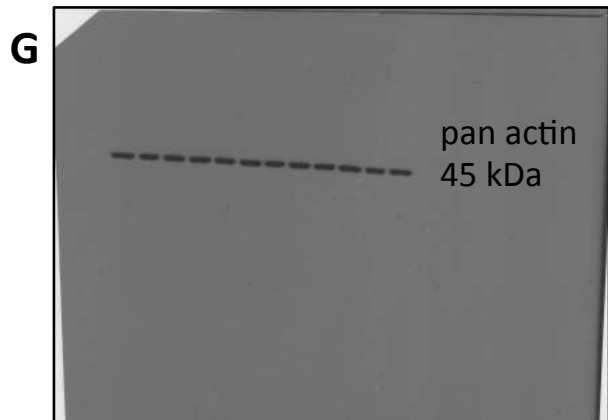

Supplement: S6 Fig — (A) phospho-p38, (B) total p38, (C) phospho-ERK1/2, (D) total ERK1/2, (E) phospho-NF-κB, (F) total NF-κB, (G) pan-actin. Order in triplicates: Control, PCS, PCS+RWJ, PCS+U0126. (PDF) [file pone.0187459.s008.pdf]
